# Polymorphism in Ionic Cocrystals Comprising Lithium Salts and l-Proline

**DOI:** 10.1021/acs.cgd.2c00172

**Published:** 2022-05-03

**Authors:** Rana Sanii, Yassin H. Andaloussi, Ewa Patyk-Kaźmierczak, Michael J. Zaworotko

**Affiliations:** † Department of Chemical Sciences and Bernal Institute, University of Limerick, Co., Limerick V94T9PX, Ireland; ‡ Department of Materials Chemistry, Faculty of Chemistry, Adam Mickiewicz University in Poznań, Uniwersytetu Poznańskiego 8, Poznań 61-614, Poland

## Abstract

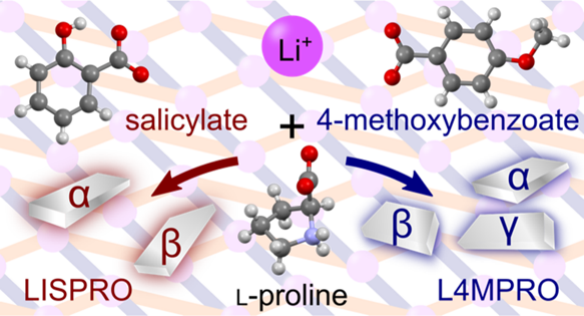

The occurrence of polymorphism in ionic cocrystals formed by two lithium salts, lithium salicylate (LIS) and lithium 4-methoxybenzoate (L4M), and l-proline (PRO) has been investigated. The previously reported monoclinic form of the 1:1 cocrystal of LIS and PRO, LISPRO(α), and a new thermodynamically stable orthorhombic polymorph, LISPRO(β), were prepared and characterized. The two polymorphs form square grid, sql, topology coordination networks and differ mainly in the conformation of the salicylate ions and positioning of the sql nets. LISPRO(α) was observed to transform to LISPRO(β) under slurry conditions. The 1:1 ionic cocrystal of L4M and PRO (L4MPRO) was found to form three polymorphs. Apart from the previously reported orthorhombic crystal form, L4MPRO(α), two new monoclinic crystal forms, L4MPRO(β) and L4MPRO(γ), were obtained by modifying crystallization conditions. The new polymorphs were found to be metastable, undergoing transformations to L4MPRO(α) upon exposure to humidity. Experimental conditions that induce transformations between the polymorphs of LISPRO and L4MPRO are detailed, and the structural differences between the polymorphs are discussed in the broader context of polymorphism.

## Abstract 2

Polymorphism in ionic cocrystals of lithium salicylate (LIS) and lithium 4-methoxybenzoate (L4M) with l-proline (PRO) as a coformer was investigated, affording a more thermodynamically stable polymorph of lithium salicylate l-proline [LISPRO(β)] and two metastable polymorphs of lithium 4-methoxybenzoate l-proline (L4MPRO). The crystal structures and experimental conditions that enabled transformations between polymorphs are detailed.

## 1 Introduction

Lithium salts have a long history of therapeutic use in the treatment of bipolar disorder,^1^ amnestic mild cognitive impairment,^2^ Alzheimer’s disease,^3^ and mania.^4^ More recently, crystal engineering studies of lithium-containing compounds^5,6^ have been driven by a desire to improve the narrow therapeutic window of FDA-approved lithium drug products such as lithium carbonate and lithium citrate. This therapeutic window hinders the use of lithium as a treatment as it requires regular and intensive evaluation of blood, thyroid, and kidney function to monitor the effects of lithium toxicity. Lithium toxicity can cause loss of consciousness, epileptic seizures, constipation, and hyperreflexia, which can negatively impact a patient’s compliance with treatment.^7^ Despite these drawbacks, lithium remains the most viable option for several indications, so formulation strategies to enable controlled delivery, reduce dose frequency, and decrease peak serum lithium concentrations have been studied to minimize side effects. In this context, our group^6^ reported the lithium salicylate (LIS) l-proline ionic cocrystal, LISPRO, which is currently being developed under the trade name LiProSal. Importantly, LISPRO was found to exhibit lower peak serum lithium concentration and longer retention time in the blood stream than lithium carbonate. In principle, LISPRO should therefore reduce lithium toxicity and enable a less-demanding dosage schedule. Additional advantages and mechanisms of action for LISPRO have been elaborated by Habib et al.^8−11^ The related ionic cocrystal (ICCs), lithium 4-methoxybenzoate l-proline (L4MPRO), disclosed in a patent,^12^ offers similar promise as a therapeutic. As noted by a reviewer of this article, there is some ambiguity as to whether ICCs of the type studied herein should be referred to as coordination polymers. In our opinion, these terms are not mutually exclusive and the context would affect which term is adopted. From the perspective of pharmaceutical science, the term ionic cocrystal would therefore be appropriate.^13,14^

Despite the evident pharmacological benefits of lithium-based ICCs,^15^ this group of compounds remains understudied in the literature from a crystal engineering perspective.^16^ Forty-seven examples^17−20^ of natural amino acid-based lithium ICCs are archived in the Cambridge Structural Database^21^ (CSD; ConQuest^22^ 2020.3.0, CSD v5.42 May 2021, *R* factor ≤10.0%, only single-crystal structures), and only one case of lithium ICC polymorphism has been reported thus far.^5^ Polymorphism,^23−26^ the phenomenon in which a chemical compound exhibits different crystal structures, is long known but understudied outside pharmaceutical compounds. In this context, the United States Food and Drug Administration (FDA) has recognized the relevance of polymorphism to drug products and defined polymorphism as “the ability of a substance to exist in at least two crystalline forms with different crystal packing arrangements and/or conformations of molecules in the crystal lattice”.^27^

The term “polymorphism” was first used in 1822 by Mitscherlich when he noted different physical and chemical properties of crystals of arsenates and phosphates.^28^ However, in 1809, Humphrey Davy noted that both diamond and graphite were made of carbon, with the only difference being the arrangement of carbon atoms in the solid phase.^29^ The first report of a polymorphic organic compound dates back to 1832 with the discovery of a polymorph of benzamide by Liebig and Wöhler, which was obtained during the heat–cool crystallization of an aqueous solution of benzamide.^30^

The primary reason that the pharmaceutical industry is interested in studying polymorphs is that most active pharmaceutical ingredients (APIs) are delivered as crystalline drug substances and polymorphs can vary in their physical, chemical, mechanical, and biopharmaceutical properties as well as their stability, thereby impacting utility.^31,32^ Further, if the polymorphic landscape of a compound is not understood, the unexpected emergence of an unwanted solid form, or conversion between polymorphs, can result in negative consequences, as exemplified by Ritonavir.^33^

Whereas differences in polymorphic properties such as aqueous solubility and melting point are typically negligible,^34−36^ screening for new solid forms of drug substances and studying their stability has become a routine and required aspect at the pre-clinical stage of drug development.^37−39^ Such screening can include the isolation and study of multicomponent crystals such as solvates, hydrates, and pharmaceutical cocrystals^40−42^ (cocrystals in which at least one of the coformers is an API and the other coformer is a pharmaceutically acceptable molecule or ion).^43^ Pharmaceutical cocrystals, which can offer very different physicochemical properties and a diverse range of crystal forms without compromising therapeutic benefits,^44−46^ have been bolstered by the FDA and EMA releasing regulatory guidelines for industry on the use of pharmaceutical cocrystals.^47,48^ Indeed, the number of marketed drug products based on APIs that are pharmaceutical cocrystals has increased in recent years,^49^ with four new drug products approved between 2014 and 2017.^49^ In addition, a new pharmaceutical ICC is the API in Seglentis, which received FDA approval in October 2021.^50,51^

Whereas polymorphism in cocrystals^46,52−54^ has been reported, it remains understudied in the context of pharmaceutical cocrystals. Herein, we report the synthesis and characterization of polymorphs of ICCs of LIS and L4M with l-proline. Only one form of each ICC had been reported prior to this study, LISPRO(α)^6^ and L4MPRO(α).^55^

## 2 Experimental Section

### 2.1 General Aspects

Reagents were purchased from Sigma-Aldrich (l-proline, lithium hydroxide, and 4-methoxybenzoic acid) or Alfa Aesar (LIS) and used without further purification. Powder X-ray diffraction (PXRD) data were measured on an Empyrean diffractometer (PANanalytical, Philips) equipped with a PIXcel^3D^ detector using a Cu Kα radiation source in reflection geometry. Thermogravimetric analyses were performed on a TA Instrument Q50 TG. Differential scanning calorimetry (DSC) analyses were carried out on a TA Instrument DSC Q20. Fourier transform infrared (FTIR) spectra were collected on a PerkinElmer Spectrum 100 spectrometer with a Universal ATR accessory.

### 2.2 Single-Crystal X-ray Data Collection and Structure Determination

Crystals of LISPRO (α and β) and L4MPRO (α, β, and γ) were examined under a microscope, and suitable single crystals were selected for single-crystal X-ray diffraction (SCXRD) analysis. Single-crystal structures were determined at RT or 100 K using either Mo K_α_ (λ = 0.71073 Å) or Cu K_α_ (λ = 1.5418 Å) radiation on a Bruker D8 Quest fixed-chi diffractometer equipped with a Bruker APEX-II CCD detector and a nitrogen-flow Oxford Cryosystem attachment. Data was indexed, integrated, and scaled in APEX3.^56^ Absorption corrections were performed by the multi-scan or numerical method using SADABS^57^ or TWINABS.^58^ Space groups were determined using XPREP^59^ as implemented in APEX3. The SHELX-2014 program package, implemented in OLEX2^60^ v1.2.8 or v1.3.0, was used for structure solution and refinement. Structures were solved using the intrinsic phasing method (SHELXT)^61^ and refined with SHELXL^62^ using the least-squares method. All non-hydrogen atoms were refined anisotropically. Hydrogen atoms were placed in calculated positions from the molecular geometry and assigned isotropic thermal parameters that depended on the equivalent displacement parameters of their carriers. Crystal data were deposited with the Cambridge Crystallographic Data Centre (CCDC 2116205–2116210 and 2142069–2142072). Selected crystallographic data and refinement parameters for the crystal structures refined from data collected at 100 K are given in Table 1. For the detailed crystallographic information associated with this work, including crystallographic data for structures collected at room temperature, please refer to Tables S1–S14 in the Supporting Information.

**Table 1:** Selected Crystallographic Data and Structure Refinement Parameters

compound abvr.	LISPRO(α)	LISPRO(β)	L4MPRO(α)	L4MPRO(β)	L4MPRO(γ)
compound	Li(salicylate)(l-proline) (α)	Li(salicylate)(l-proline) (β)	Li(4-methoxybenzoate)(l-proline) (α)	Li(4-methoxybenzoate)(l-proline) (β)	Li(4-methoxybenzoate)(l-proline) (γ)
formula	C_12_H_14_LiNO_5_	C_12_H_14_LiNO_5_	C_13_H_16_LiNO_5_	C_13_H_16_LiNO_5_	C_13_H_16_LiNO_5_
MW (g·mol^–1^)	259.18	259.18	273.21	273.21	273.21
*T* (K)	100(2)	100(2)	100(2)	100(2)	100(2)
crystal system	monoclinic	orthorhombic	orthorhombic	monoclinic	monoclinic
space group	*P*2_1_	*P*2_1_2_1_2_1_	*P*2_1_2_1_2_1_	*P*2_1_	*P*2_1_
*a* (Å)	10.3035(8)	10.2736(4)	5.3718(1)	5.4154(2)	10.4879(13)
*b* (Å)	10.1046(8)	10.1049(3)	9.1760(3)	8.7562(4)	10.0527(12)
*c* (Å)	11.9572(11)	23.9524(9)	26.5432(7)	14.1554(7)	13.2148(16)
α (deg)	90.00	90.00	90.00	90.00	90.00
β (deg)	94.253(6)	90.00	90.00	95.493(2)	107.653(4)
γ (deg)	90.00	90.00	90.00	90.00	90.00
*V* (Å^3^)	1241.47(18)	2486.59(15)	1308.36(6)	668.14(5)	1327.7(3)
ρ_calc_ (g·cm^–3^)	1.387	1.385	1.387	1.358	1.367
*Z*, *Z′*	4, 2	8, 2	4, 1	2, 1	4, 2
observed reflections	3541	4383	3291	2947	5272
[*I* > 2σ(*I*)]					
*R*_1_, w*R*_2_ [*I* > 2σ(*I*)]	0.0751, 0.1972	0.0507, 0.1267	0.0338, 0.0754	0.0336, 0.0770	0.0642, 0.1556
*R*_1_, w*R*_2_ (all data)	0.0830, 0.2054	0.0551, 0.1300	0.0415, 0.0785	0.0412, 0.0801	0.0890, 0.1677
goodness-of-fit on *F*^2^	1.055	1.120	1.040	1.037	1.049
*R*_int_ value (%)	5.65	7.63	3.37	3.83	5.79

### 2.3 Syntheses of Lithium ICCs

#### 2.3.1 LISPRO(α)

**Figure 1:**
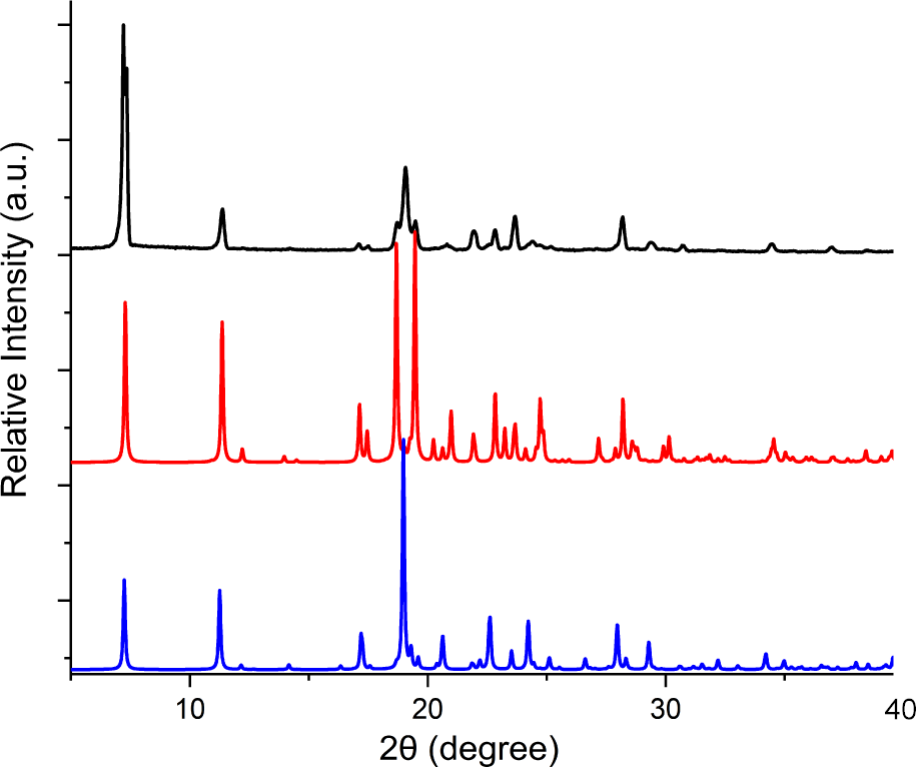
Experimental PXRD patterns of LISPRO(α) (black) overlayed with calculated PXRDs of LISPRO(α) (red) and LISPRO(β) (blue) from crystals collected at RT. The presence of a peak at 19.0° indicates the presence of LISPRO(β) in samples of LISPRO(α) in what are otherwise similar PXRD patterns.

LIS (144 mg, 1 mmol) and l-proline (115 mg, 1 mmol) were dissolved in 2 mL H_2_O and heated to 70 °C for ca. 1 h in an open vial to allow solvent evaporation until crystals had formed. The initially formed crystals were harvested and stored under oil. These crystals were found to be of LISPRO(α), as verified by SCXRD; however, a PXRD of the crystals was found to be a mixture of LISPRO(α) and LISPRO(β) (Figure 1).

#### 2.3.2 LISPRO(β)

A 1:2 water slurry of LIS (144 mg, 1 mmol) and l-proline (230 mg, 2 mmol) in 1.5 mL H_2_O was conducted under ambient conditions for 24 h. The resulting powder was filtered and air-dried. Recrystallization experiments using various solvents such as MeOH, EtOH, MeCN, and i-PrOH were conducted in order to obtain single crystals suitable for SCXRD. Then, 14 mg of the sample obtained from slurry was dissolved in 1.5 mL EtOH in a loosely covered vial and left to undergo slow evaporation. Colorless needle-shaped crystals of LISPRO(β) were obtained after 3 days.

#### 2.3.3 L4MPRO(α)

L4M was prepared by grinding lithium hydroxide (300 mg, 12.53 mmol) and 4-methoxybenzoic acid (1.906 g, 12.53 mmol) in a mortar and pestle with 0.44 mL distilled water until a free-flowing powder was obtained (approximately 15 min) and dried in an oven at 75 °C for 20 h. A 1:10 water slurry of L4M (as-prepared, 94.5 mg, 0.598 mmol) and l-proline (688.2 mg, 5.978 mmol) in 0.5 mL H_2_O was conducted at ambient conditions for 24 h, affording colorless needle-shaped crystals of L4MPRO(α), as verified by a PXRD.

Seeding-assisted mechanochemical synthesis of L4MPRO(α) was performed by grinding L4M (as-prepared, 100 mg, 633 mmol, 1 equiv) and l-proline (72.8 mg, 633 mmol, 1 equiv) with 5 mol % L4MPRO(α) and 0.04 mL distilled water in a mortar and pestle until a free-flowing powder was formed (approximately 15 min). A PXRD performed on the resultant powder revealed it to be a mixture of L4MPRO(α) and L4MPRO(β). Then, 30 mg of this mixture (0.11 mmol) was slurried in 0.15 mL EtOH under ambient conditions for 3 days, resulting in L4MPRO(α), as confirmed by PXRD.

#### 2.3.4 L4MPRO(β)

Colorless plate crystals were prepared by dissolving L4M (as-prepared, 158 mg, 1 mmol) and l-proline (575 mg, 5 mmol) in 2 mL H_2_O and heating to 70 °C for ca. 1 h in an open vial to allow solvent evaporation until crystals had formed. The initially formed crystals were harvested and stored under oil. These crystals were determined to be L4MPRO(β) via SCXRD. A powder sample was prepared with L4M (as-prepared, 300 mg, 1.9 mmol) ground in a mortar and pestle with l-proline (218.5 mg, 1.9 mmol) and 0.11 mL distilled water until a free-flowing powder was formed (approximately 15 min). Alternatively, L4MPRO(β) was prepared by slurrying L4M (as-prepared, 50 mg, 0.316 mmol) and l-proline (36.4 mg, 0.316 mmol) for 24 h in 0.52 mL EtOH, ethyl acetate, or acetone or 0.17 mL dimethylformamide (DMF), chloroform, or tetrahydrofuran. The slurry procedure with 0.17 mL MeCN yielded a mixture of L4MPRO(β) and L4MPRO(γ).

#### 2.3.5 L4MPRO(γ)

L4M (as-prepared, 50 mg, 0.316 mmol) and l-proline (36.4 mg, 0.316 mmol) were dissolved in a heated mixture of 4 mL MeOH and 0.1 mL H_2_O. The vial was loosely covered, and after 24 h, large plate-shaped crystals of L4MPRO(γ) formed along the vial walls. A powdered sample of L4MPRO(γ) was formed by grinding L4M (as-prepared, 300 mg, 1.9 mmol) and l-proline (218.5 mg, 1.9 mmol) with 0.32 mL dimethyl sulfoxide (DMSO) until a homogenous powder was formed. Additionally, L4MPRO(γ) was obtained by slurrying L4M (as-prepared, 50 mg, 0.316 mmol) and l-proline (36.4 mg, 0.316 mmol) in either 0.52 mL MeOH or i-PrOH for 24 h. A similar slurry procedure with 0.17 mL MeCN yielded a mixture of L4MPRO(β) and L4MPRO(γ).

### 2.4 Stability Tests

In order to explore the effect of humidity on lithium ICCs, accelerated stability testing under conditions used in the pharmaceutical industry (40 °C and 75% RH) were employed.^63^ The synthesized ICCs were subjected to such stability testing by placing 30 mg of each compound in a glass vial loosely covered with aluminum foil in a humidity chamber at 75% RH and 40 °C. Aliquots were removed after 14 days, and PXRD data were collected.

### 2.5 Purity Confirmation

The purity of the ICCs was assessed by PXRD and DSC. Comparisons of the experimental PXRD patterns of as-prepared ICCs with PXRD patterns calculated based on crystal structures are presented in Figures S1, S2, and S7–S10.

## 3 Results and Discussion

### 3.1 LISPRO

Several attempts to synthesize the previously reported form of LISPRO^6^ through slurrying afforded PXRD patterns (Figure S2) that did not match the calculated PXRD pattern of the previously reported form of LISPRO, herein called LISPRO(α). Interestingly, the PXRD pattern did resemble the experimental PXRD reported by Smith et al.^6^ Single-crystal analysis of an ethanol-recrystallized sample of LISPRO revealed a new polymorph, LISPRO(β). The experimental PXRD of the as-prepared LISPRO and the calculated PXRD patterns of the α and β polymorphs are overlaid in Figures 1, S1, and S2. The experimental PXRD pattern of LISPRO(β) is in good agreement with that calculated from the crystal structure of LISPRO(β). Subsequent attempts to obtain a pure sample of LISPRO(α), either by rapid solvent evaporation or by slurrying, failed, with all samples containing at least some LISPRO(β) according to PXRD data (Figures 1 and S1). A close inspection of the experimental PXRD of the first report of LISPRO^6^ hints at this same outcome, with a mixture of peaks consistent with LISPRO(α) and LISPRO(β). This precluded us from conducting a 50:50 slurry of the two LISPRO polymorphs. Rather, a mixture of LISPRO(α) and LISPRO(β) obtained from heating–cooling was slurried in EtOH for 24 h. This experiment afforded pure LISPRO(β), indicating that it is more stable than LISPRO(α) (Figure S27) under these conditions. No polymorphic transformation was observed when crystals of LISPRO(β) were exposed to accelerated stability testing conditions (75% RH, 40 °C) for 14 days (Figure S30). Long-term stability studies of LISPRO(β) under ambient conditions confirmed solid-form stability for at least 6 months.

### 3.2 L4MPRO

**Figure 2:**
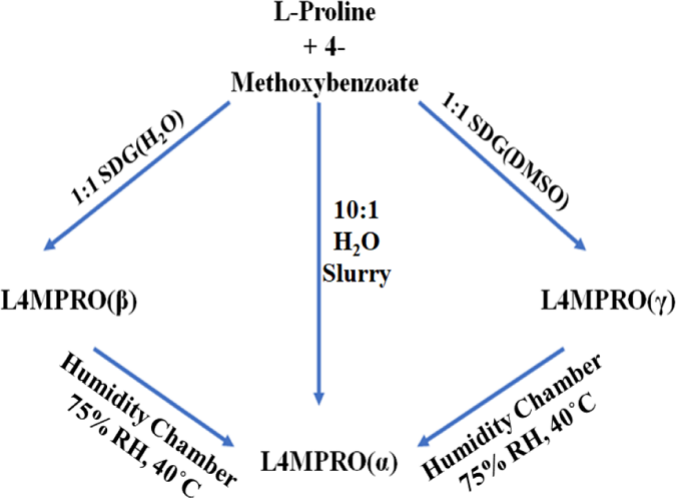
Scheme showing transformations between polymorphs of the L4MPRO ICCs and selected methods for the synthesis of the respective L4MPRO forms.

As illustrated in Figure 2, L4MPRO was found to exist in three polymorphic forms, as characterized by FTIR, PXRD, and SCXRD (Figures S7–S10, S16). The previously reported form, L4MPRO(α),^55^ was obtained as single crystals from the H_2_O slurry of l-proline and L4M in a 10:1 molar ratio. Attempts to prepare L4MPRO(α) through SDG^64^ (also known as liquid-assisted grinding^65,66^) failed; a 1:1 water grind yielded L4MPRO(β), while a 1:1 DMSO grind afforded a third polymorph, L4MPRO(γ). The 1:1 slurry experiments also failed to produce L4MPRO(α), with most solvents, for example, ethanol and DMF, resulting in L4MPRO(β), while MeOH and i-PrOH afforded L4MPRO(γ) (Figure S11). Similar experiments conducted in water generally failed to produce L4MPRO, resulting in L4M.

That L4MPRO(α) did not form under slurry conditions would typically indicate relative instability compared to the other polymorphs; however, when L4MPRO(β) and (γ) were exposed to accelerated stability-testing conditions (75% RH, 40 °C) for 14 days, transformation to L4MPRO(α) occurred (Figures S31–S33). Seeding the as-formed L4MPRO(α) crystals into a 1:1 water-based SDG synthesis of LM4PRO resulted in a powder comprising L4MPRO(α) with only minor quantities of L4MPRO(β) (Figure S14). These results suggest that whereas LM4PRO(α) is thermodynamically favored, kinetics drives the formation of the other forms. The 50:50 slurries of either the α and β polymorphs or the α and γ polymorphs were then performed in ethanol, each resulting in transformation to the α-form (Figures S28 and S29). Long-term stability studies of the polymorphs under ambient conditions revealed that L4MPRO(α) and (β) remained stable for at least 6 months while L4MPRO(γ) converted to L4MPRO(α) (Figure S26).

The higher thermodynamic stability of L4MPRO(α) is also consistent with density.^67,68^ At room temperature, the crystal density of L4MPRO polymorphs is as follows: L4MPRO(α) > L4MPRO(β) > L4MPRO(γ) (1.358, 1.329, and 1.320 g cm^–3^, respectively), while at 100 K, the order is as follows: L4MPRO(α) > L4MPRO(γ) > L4MPRO(β) (1.387, 1.367, and 1.358 g cm^–3^, respectively).

In the case of this study, slurry-based and mechanochemical approaches were found to be complementary. Slurrying was informative with respect to relative stability and, from a screening perspective, afforded all polymorphs except for LISPRO(α), while mechanochemistry initially failed to afford L4MPRO(α). These observations further highlight the benefit of using multiple screening approaches for cocrystal discovery.^69^

### 3.3 Crystal Structures from SCXRD

In this section, we refer to crystal structures determined from data collected at 100 K unless stated otherwise.

#### 3.3.1 LIS l-Proline, LISPRO(β)

**Figure 3:**
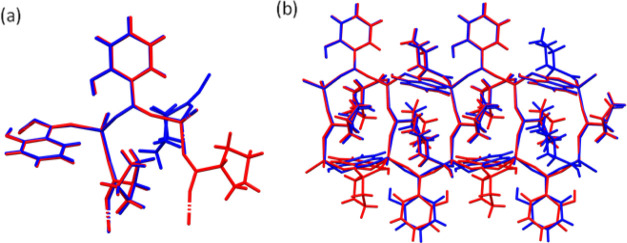
Overlay of the (a) asymmetric unit and (b) fragment of the square grid of LISPRO(α) (red) and LISPRO(β) (blue). The overlay was prepared using the structure overlay tool in mercury, for carboxylate groups and lithium ions in LISPRO forms. For clarity, only the dominant disorder component for both polymorphs is shown.

LISPRO(β) crystallized in the orthorhombic space group *P*2_1_2_1_2_1_. The unit cell was found to contain eight lithium cations, eight salicylate anions, and eight l-proline molecules, with two formula units [Li(Pro)(Sal)] in the asymmetric unit (*Z*′ = 2). Within the asymmetric unit, one salicylate and one l-proline molecule is disordered, with the aromatic ring of the salicylate molecules rotated to allow intramolecular hydrogen bonding with either of its carboxylate group oxygen atoms. The major components of the salicylate and l-proline disorder have site occupancies of 0.833(8) and 0.802(11), respectively. Lithium cations exhibit a tetrahedral coordination geometry, being linked by four bridging carboxylate moieties, two from salicylate and two from l-proline. The resulting structure is a zigzag square grid network propagating in the (001) plane (Figures 3 and S3–S5; for the Li–O bond lengths, please see Tables S5 and S6). The amine groups of l-proline zwitterions form hydrogen bonds within the square grids to the carboxylate oxygen atoms of l-proline with a distance of 2.733 (4) Å or 2.779(4) Å and to the carboxylate oxygen of a neighboring salicylate with a distance of 2.830(4) Å or 3.081(8) Å (for the major component). Additional hydrogen bonds are formed by salicylate ions between the hydroxyl group and the carboxylate group, with distances of 2.593(8) and 2.583(4) Å. Salicylate and l-proline molecules form square grids that alternate in their orientation. The l-proline zwitterions alternately point above and below the walls of the zigzag grid, while salicylate ions alternate between pointing near-parallel or at an angle to the walls, enabling the close packing of adjacent square grids.

#### 3.3.2 LIS l-Proline, LISPRO (α)

To allow appropriate comparison of structures, SCXRD data for LISPRO(α) was recollected at 100 K. As previously reported,^6^ LISPRO(α) crystallized in *P*2_1_ with a unit cell containing four lithium cations, four salicylate anions, and four l-proline zwitterions. There are two formula units [Li(Pro)(Sal)] in the asymmetric unit (*Z*′ = 2). One salicylate and one l-proline molecule of the asymmetric unit show a disorder similar to that observed for LISPRO(β). The major components of the salicylate and l-proline disorder have site occupancies of 0.761(15) and 0.55(2), respectively. Lithium cations exhibit a tetrahedral coordination geometry through bonding to salicylate anions and l-proline molecules via carboxylate groups, resulting in a square grid propagating in the (001) plane (for the Li–O bond lengths, please see Tables S7 and S8). Amine groups of l-proline zwitterions form hydrogen bonds within the square grids to the carboxylate oxygen of l-proline with a distance of 2.752(7) or 2.773(7) Å and to the carboxylate oxygen of a neighboring salicylate with a distance of 2.843(8) or 3.125(15) Å. Additional hydrogen bonds form between the hydroxyl and carboxylate groups of salicylate molecules, with distances of 2.610(9) and 2.557(15) Å (for the major component). Salicylate and l-proline molecules in the square grids alternate in their arrangement relative to the grid, with l-proline zwitterions alternating between pointing above and below the walls of the grid and salicylate ions positioned either in a parallel manner or at an angle, resulting in a similar structure to LISPRO(β).

#### 3.3.3 LISPRO Polymorph Comparison

**Figure 4:**
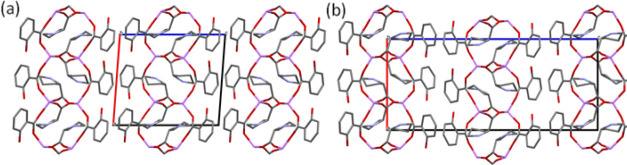
Crystal packing in (a) LISPRO(α) and (b) LISPRO(β) shown along the [010] direction. For clarity, only the major components of the disorder are shown and hydrogen atoms have been removed.

**Figure 5:**
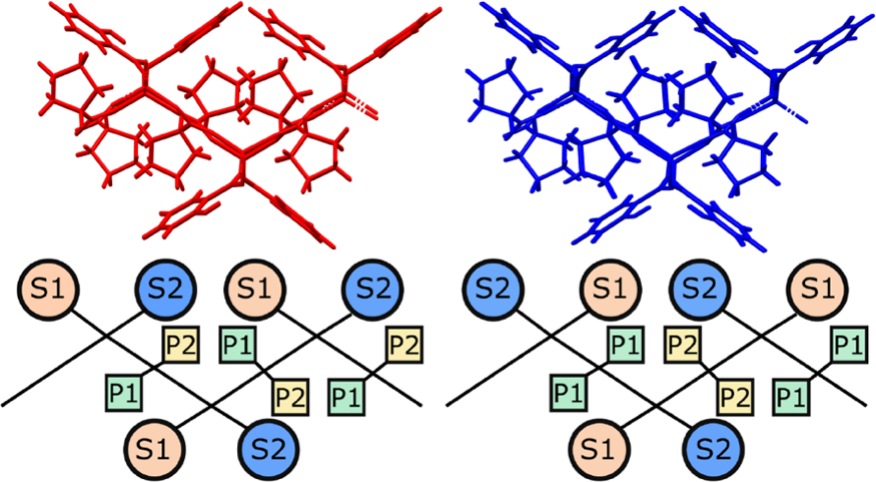
Fragment of the square grid network in LISPRO polymorphs α (red) and β (blue) shown along the direction [100] (top); schematic representation of the relative arrangement of symmetrically independent l-proline (P1/P2) and salicylate (S1/S2) moieties in the grid (bottom); and the disordered and non-disordered moieties labeled 1 and 2, respectively.

The similarity of PXRD patterns of the LISPRO polymorphs can be explained by the closely related crystal structures of LISPRO α and β, despite differences in the crystal symmetry (*P*2_1_ and *P*2_1_2_1_2_1_ space groups, respectively). The volume of the unit cell is doubled in the case of the β polymorph due to the different space group and doubling of the unit-cell parameter *c*. In both LISPRO structures, one l-proline molecule is disordered between two positions on the C4 methylene carbon, pointing either above or below the plane of the 5-membered ring. In addition, the structure of each polymorph contains one symmetrically independent salicylate ion that shows the disorder of its aromatic ring, with major and minor components. In the structure of LISPRO(α), the hydroxyl groups of disordered salicylate ions (the major component) and a second symmetrically independent salicylate orient in opposite directions within the same 2D net, that is, in the [1̅00] and [100] directions, respectively. Meanwhile, in LISPRO(β), the hydroxyl groups of both symmetrically independent salicylates are positioned in the same direction [1̅00] within the nets (considering the major component of the disorder), with adjacent 2D nets having hydroxyl groups oriented in the opposite direction [100] (Figure 4). Conformational differences between the square grids of LISPRO(α) and (β) can be noticed when the structures are overlaid (Figures 3–5). Alongside structural differences within the square grids, the α and β forms of LISPRO differ in the relative positioning of the grids. This displacement of the grids can be expressed by the angle between symmetry-related lithium cations of adjacent 2D grids stacked in the [001] direction. For LISPRO(α), this angle is 180°, while for LISPRO(β), it is 170.5° (Figure S6).

The structural similarities between LISPRO(α) and LISPRO(β) mean that there is a small difference in the calculated densities. At 100 K, the α polymorph is the denser phase with a calculated density of 1.387 g cm^–3^ compared to 1.385 g cm^–3^ for LISPRO(β). At RT (i.e., conditions at which the stability experiments were conducted), the relative density is preserved, with β being less dense (1.334 g cm^–3^) than α (1.350 g cm^–3^). However, it should be noted that the quality of the X-ray diffraction data at room temperature was affected by the poor diffracting properties of LISPRO crystals, reducing the accuracy of the unit-cell parameters and the calculated crystal density.

#### 3.3.4 L4M L-Proline, L4MPRO(α)

**Figure 6:**
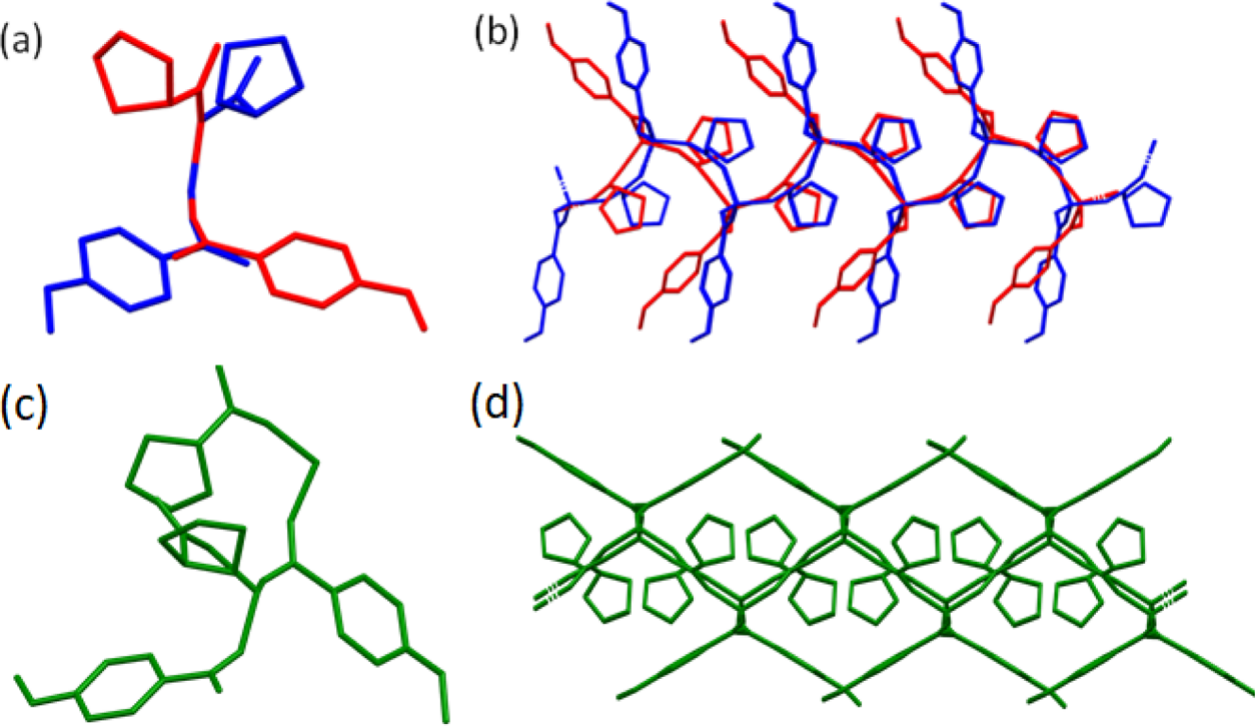
Overlay of the asymmetric unit (a) and fragment of a square grid (b) in L4MPRO polymorphs α and β, shown in red and blue, respectively, viewed along the [100] direction and formed by overlaying lithium ions. Asymmetric unit (c) and square grid (d) of L4MPRO(γ) (green; viewed along the [100] direction) have been shown separately. For clarity, hydrogen atoms have been removed.

L4MPRO(α) crystallized in the orthorhombic space group *P*2_1_2_1_2_1_ with *Z*′ = 1. SCXRD revealed that L4MPRO(α) contains four lithium cations, four 4-methoxybenzoate anions, and four l-proline molecules in the unit cell, with the asymmetric unit comprising one molecule of l-proline, one 4-methoxybenzoate anion, and one lithium cation (Figure 6). Each lithium cation is tetrahedrally coordinated and bridged by four carboxylate moieties, two from l-proline and two from 4-methoxybenzoate, to form zigzag square grid networks in the (001) plane (for Li–O bond lengths, please see Tables S9 and S10). The lithium cations are bridged in the [100] direction by 4-methoxybenzoate ions and in the [011] and [011̅] directions by l-proline zwitterions, in an alternating manner, resulting in a wave-like square grid net. The protonated amine groups of l-proline zwitterions are H-bonded to a carboxylate oxygen of a neighboring l-proline with a distance of 2.843(2) Å and to the carboxylate oxygen of a neighboring 4-methoxybenzoate anion with a distance of 2.880(2) Å. The folding of the square grid can be assessed by the Li–C(carbonyl)–Li angle, as measured between two lithium cations linked by an l-proline zwitterion, and the Li–Li–Li angle between consecutive lithium cations arranged in a zigzag manner in the [010] direction. For the square grid of L4MPRO(α), these angles are 149.5(1) and 103.9(1)°, respectively. The 4-methoxybenzoate moieties lie nearly parallel to the square grid walls at an angle of 6.5(7)°, as measured between the plane of parallel C6–C7 bonds of 4-methoxybenzoate ions and the plane formed by lithium ions in one square of the square grid (Figures S12 and S13). As all 4-methoxbenzoate groups orient in the same direction within individual square grids, a gap is created between each “rung” of the wave-like net and is filled by a 4-methoxybenzoate of an adjacent grid (symmetrically related through a twofold screw axis along [100]).

#### 3.3.5 L4MPRO(β)

L4MPRO(β) crystallized in the monoclinic space group *P*2_1_ with *Z*′ = 1. SCXRD revealed that L4MPRO(β) contains two lithium cations, two 4-methoxybenzoate anions, and two l-proline molecules in the unit cell, with the asymmetric unit comprising one molecule of l-proline, one 4-methoxybenzoate anion, and one lithium cation. Like L4MPRO(α), each lithium cation is tetrahedrally coordinated and bridged by four carboxylate moieties, two from l-proline and two from 4-methoxybenzoate anions, to form square grid networks in a staircase-like arrangement along the (001) plane (for the Li–O bond lengths, please see Tables S11 and S12). Within the square grid, protonated amine groups of l-proline zwitterions form H-bonds to a carboxylate oxygen of a neighboring l-proline with a distance of 2.790(2) Å and to a carboxylate oxygen of a neighboring 4-methoxybenzoate with a distance of 2.761(2) Å. The Li–C(carbonyl)–Li angle between two lithium cations linked by an l-proline zwitterion and the Li–Li–Li angle between consecutive lithium cations arranged in a zigzag manner in the [010] direction are 134.2(1) and 105.8(1)°, respectively. Unlike L4MPRO(α), the 4-methoxybenzoate groups are not aligned with the walls of the square grid, with an angle of 43.3(9)° between the plane of parallel C6–C7 bonds of 4-methoxybenzoate ions and the plane formed by four lithium ions (Figures S12 and S13). Similar to L4MPRO(α), all 4-methoxybenzoate anions point in the same direction within a square grid, with the resulting gap being filled by 4-methoxybenzoate anions of an adjacent 2D net.

#### 3.3.6 L4MPRO(γ)

Similar to L4MPRO(β), L4MPRO(γ) crystallized in the monoclinic space group *P*2_1_, however, with *Z*′ = 2. SCXRD revealed that L4MPRO(γ) contains four lithium cations, four 4-methoxybenzoate anions, and four l-proline molecules in the unit cell with an asymmetric unit comprising two formula units. As with the α and β polymorphs, each lithium cation is tetrahedrally coordinated and bridged by four carboxylate moieties of two l-proline molecules and two 4-methoxybenzoate anions to form a square grid network in a staircase-like arrangement along the (001) plane (for Li–O bond lengths, please see Tables S13 and S14). It should be noted that due to the presence of two formula units in the asymmetric unit, only lithium cations not related by symmetry are directly bonded to each other via ligands. Moreover, neither the two l-proline molecules nor the two 4-methoxybenzoate ions that coordinate with lithium cations are symmetrically related. l-proline zwitterions form hydrogen bonds within the square grids to the carboxylate oxygen of l-proline with a distance of 2.784(5) or 2.771(5) Å and to the carboxylate oxygen of a neighboring 4-methoxybenzoate with a distance of 2.777(4) or 2.785(5) Å. The Li–C(carbonyl)–Li angle between two lithium cations linked by l-proline zwitterions is 155.6(2) or 155.5(2)°, and the Li–Li–Li angle between lithium cations in the square grids is 110.8(2)°. Unlike the α and β polymorphs, the 4-methoxybenzoate groups in the L4MPRO(γ) square grids alternate in their position with respect to the grid walls, between near-parallel orientation [with an angle of 4.5(2)° measured between the plane of parallel C6A–C7A bonds and the plane formed by the lithium cations in the grid wall)] and out-of-plane with an angle of 62.7(2)° (measured between planes of parallel C6–C7 bonds and lithium cations in the wall) (Figures S12 and S13). As a result, no cavities exist in the sides of the 2D framework, a situation similar to the LISPRO structures.

## 4 Conclusions

Cocrystallization of l-proline with LIS and L4M was studied by applying various experimental protocols that resulted in new polymorphic forms of the ICCs LISPRO and L4MPRO. Two crystal structures, LISPRO(α) and L4MPRO(α), were known prior to this study, and three new crystal forms were obtained and characterized: the β polymorph of LISPRO and the β and γ forms of L4MPRO. LISPRO(β) and L4MPRO(α) were found to be thermodynamically stable under the conditions studied. L4MPRO(α) exhibited a higher crystal density compared to polymorphs β and γ at both 100 K and RT. Pure samples of LISPRO(β) were isolated by water slurry, while all polymorphs of L4MPRO were isolated from solvent drop grinding and/or slurry. All five crystal structures analyzed herein exhibit square grid, sql, network structures. The polymorphs of LISPRO are closely related in terms of structure and density, with only minor differences with respect to the conformation of l-proline and salicylate moieties and relative positioning of the square grids. The differences between the three crystal forms of L4MPRO are more pronounced, with α and β being similar to each other in terms of the orientation of the l-proline and 4-methoxybenzoate moieties and L4MPRO(γ) being distinctly different. To conclude, whereas we herein report polymorphism in two previously reported ICCs using conventional methods, polymorphism in ICCs remains largely understudied. It is therefore premature to draw broad conclusions about the propensity of ICCs to exhibit polymorphism, and this is a subject that deserves attention through systematic studies.

## Supporting Information Available

PXRD and thermal analyses; detailed crystallographic information; FTIR and TGA comparison of LISPRO(α) and (β) and L4MPRO(α), (β), and (γ); DSC overlay of LISPRO(α) and (β) and L4MPRO(α), (β), and (γ) polymorphs; and selected geometric parameters for Li—O bonds (PDF)

## Accession Codes

CCDC 2116205–2116210 and 2142069–2142072 contain the supplementary crystallographic data for this paper. These data can be obtained free of charge via www.ccdc.cam.ac.uk/data_request/cif, or by emailing data_request@ccdc.cam.ac.uk, or by contacting The Cambridge Crystallographic Data Centre, 12 Union Road, Cambridge CB2 1EZ, UK; fax: +44 1223 336033.
