# Supplementary material for: Polymorphism in Ionic Cocrystals Comprising Lithium Salts and l-Proline
Source: Cryst Growth Des. 2022 May 3;22(6):3786–94. doi: 10.1021/acs.cgd.2c00172 (PMC9490868; doi:10.1021/acs.cgd.2c00172)
Supplement: Supplementary file 1 — cg2c00172_si_001.pdf [file cg2c00172_si_001.pdf]

Supplementary Information

Polymorphism in Ionic Cocrystals Comprising Lithium Salts and L-Proline

Rana Sanii,[a] *Yassin H. Andaloussi,*[a] *Ewa Patyk-Kaźmierczak,*[b] *and Michael J. Zaworotko*[a]*

[a] Department of Chemical Sciences and Bernal Institute, University of Limerick, Co. Limerick, Y94T9PX, Ireland.

[b] Department of Materials Chemistry, Faculty of Chemistry, Adam Mickiewicz University, Uniwersytetu Poznańskiego 8, 61-614, Poznań, Poland.

*Email: Michael.Zaworotko@ul.ie

Table of Contents

Characterisation of Compounds 3
LISPRO(α) 3
LISPRO(β) 4
LISPRO crystal structure comparisons 5
L4MPRO(α) 7
L4MPRO(β) 8
L4MPRO(γ) 9
L4MPRO Polymorph Comparisons 10
L4MPRO synthesis by slurry in different solvents 11
L4MPRO crystal structure comparisons 12
Seeding-assisted Mechanochemical Synthesis 13
FTIR spectroscopy 14
TGA 16
DSC 17
Stability testing 21
Stability after 6-month ambient storage 21
Slurry of LISPRO polymorph mixture 22
50:50 Slurry of Polymorph mixtures 23
Stability Testing Under Humidity 25
LISPRO(β) 25
L4MPRO(α) 26
L4MPRO(β) 27
L4MPRO(γ) 28
Crystallographic details 29

Characterization of Compounds

LISPRO(α)

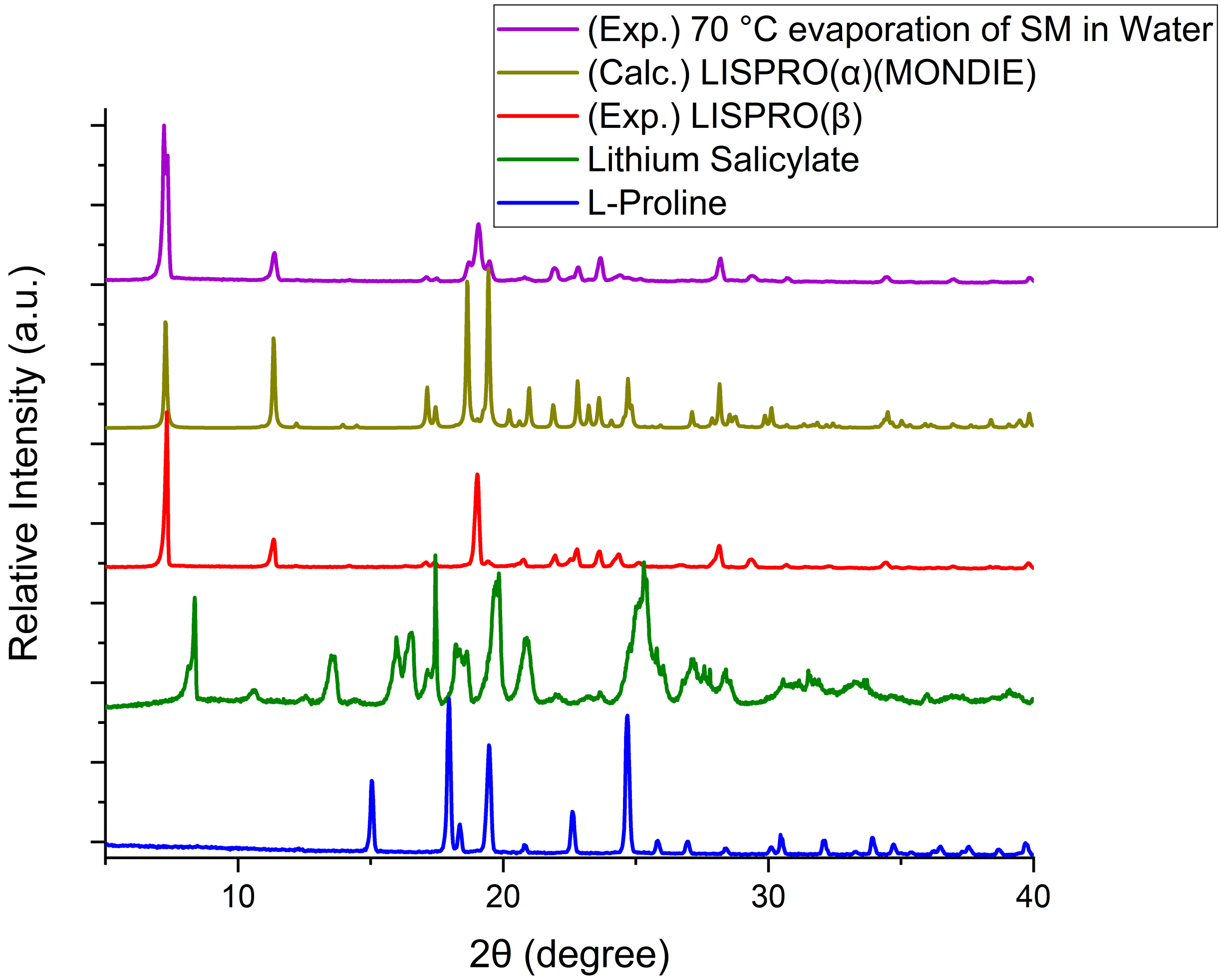

Figure S1: Experimental PXRD pattern of ground crystals from the rapid evaporation of L-proline and lithium salicylate in water at 70 °C (shown in purple) overlayed with the calculated PXRD pattern of LISPRO(α) (MONDIE) collected at 228K (shown in olive), and an experimental PXRD pattern of LISPRO(β) measured at room temperature (red). The rapid evaporation of starting materials in water (purple) leads to a powder sample containing a mixture of polymorphs, as evidenced by the PXRD pattern with peaks at 7.2°, 18.7° and 19.5° from LISPRO(α), and the peaks at 7.3° and 19.0° from LISPRO(β). Experimental PXRD patterns of lithium salicylate and L-proline are shown in green and blue, respectively.

LISPRO(β)

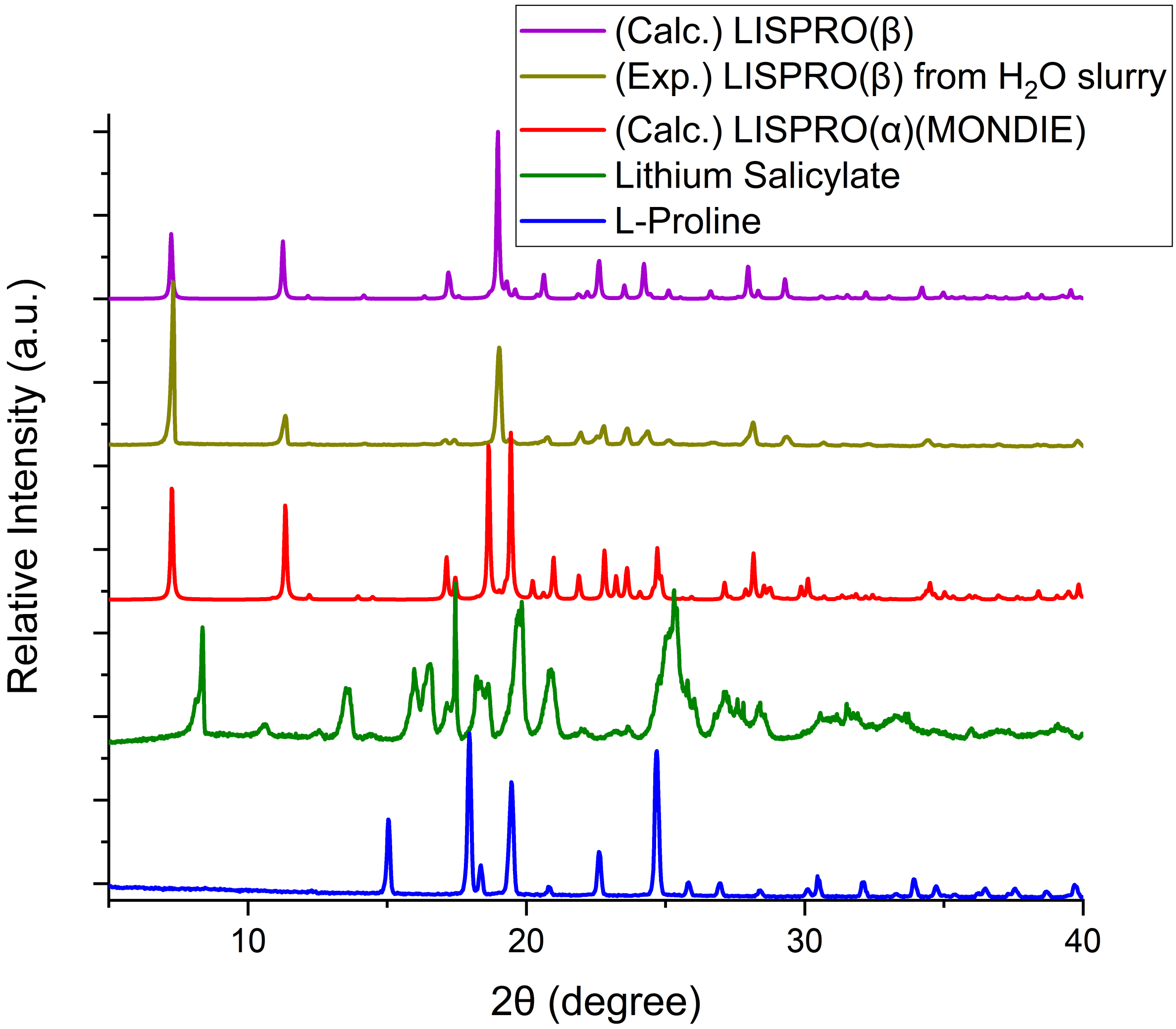

Figure S2: Calculated PXRD of LISPRO(β) (Measured at RT) in purple, compared with the PXRD of a 1:2 Lithium Salicylate: L-Proline H_2O slurry in olive showing the LISPRO(β) polymorph forming. Calculated PXRD of LISPRO(α) (from CSD entry MONDIE) in red, experimental PXRD of Lithium salicylate in green, experimental PXRD of L-Proline in Blue.

LISPRO crystal structure comparisons

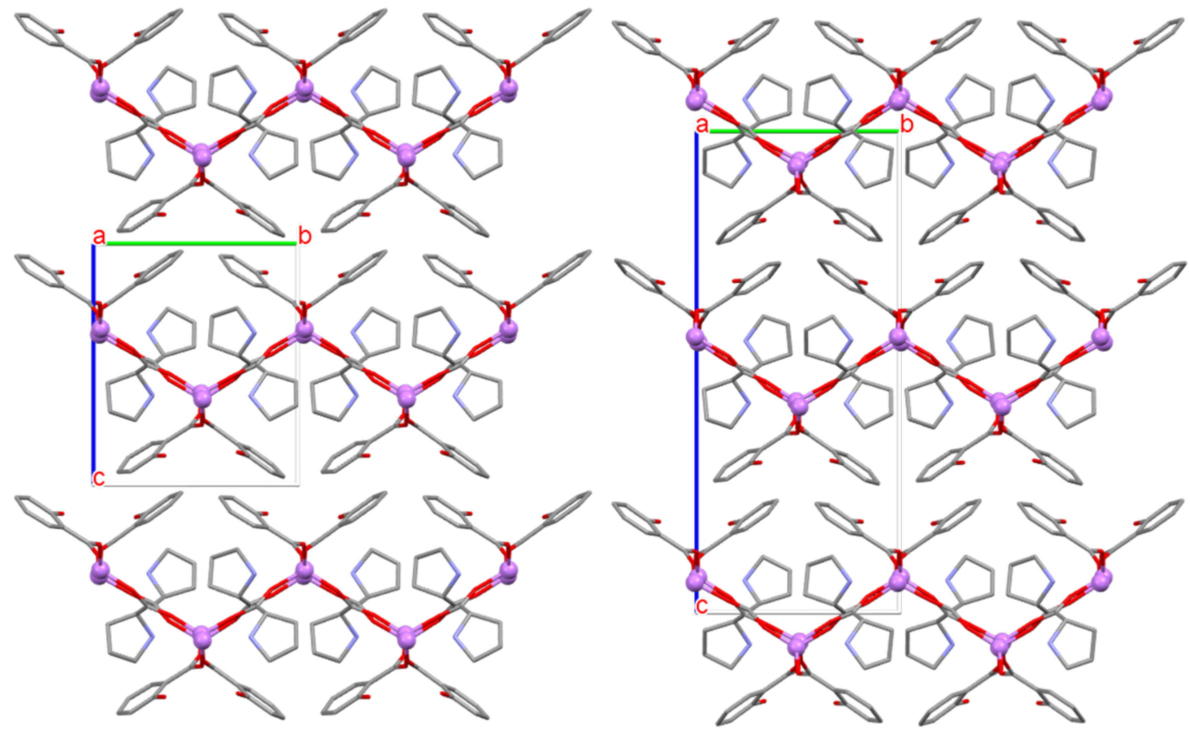

Figure S3: Crystal packing in LISPRO(α) (left) and LISPRO(β) (right) at 100 K, shown along direction [100]. For clarity, only major components of the disordered crystal structures are shown and hydrogen atoms have been removed.

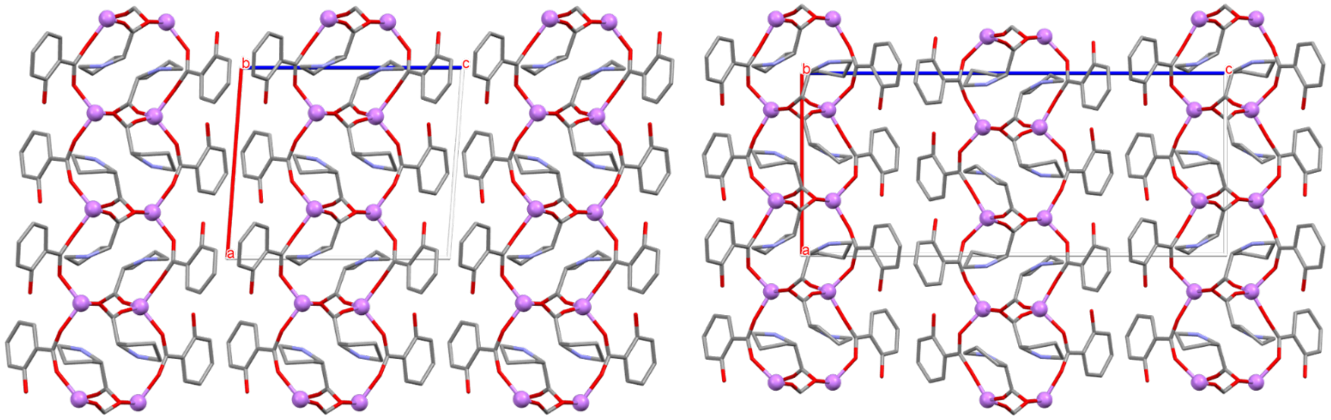

Figure S4: Crystal packing in LISPRO(α) (left) and LISPRO(β) (right) at 100 K, shown along direction [010]. For clarity, only major components of the disordered crystal structures are shown and hydrogen atoms have been removed.

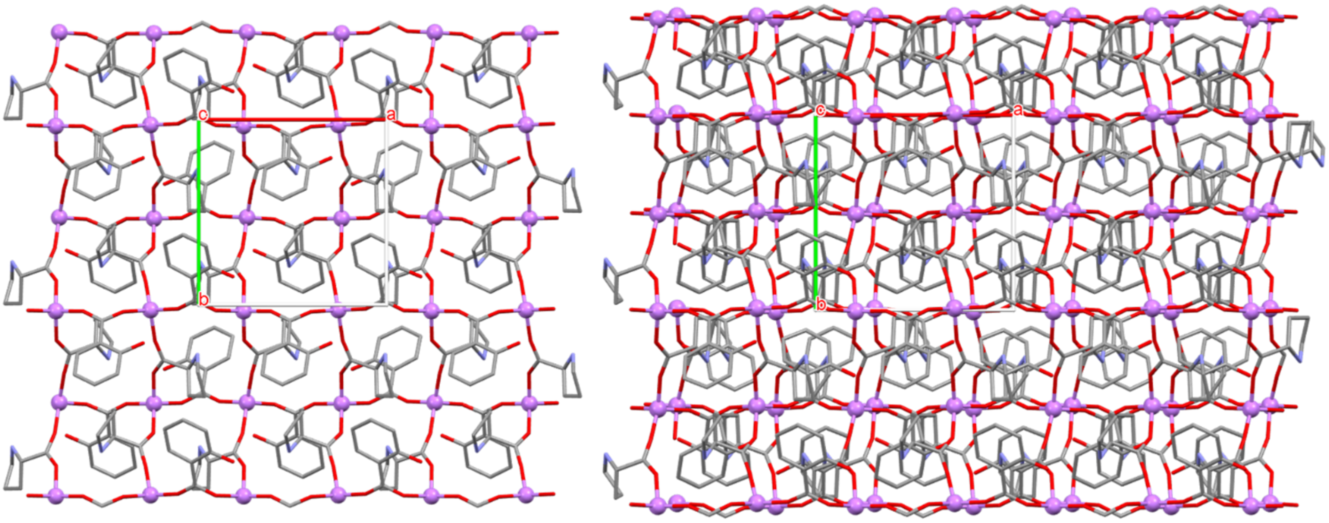

Figure S5: Crystal packing in LISPRO(α) (left) and LISPRO(β) (right) at 100 K, shown along direction [001]. For clarity, only major components of the disordered crystal structures are shown and hydrogen atoms have been removed.

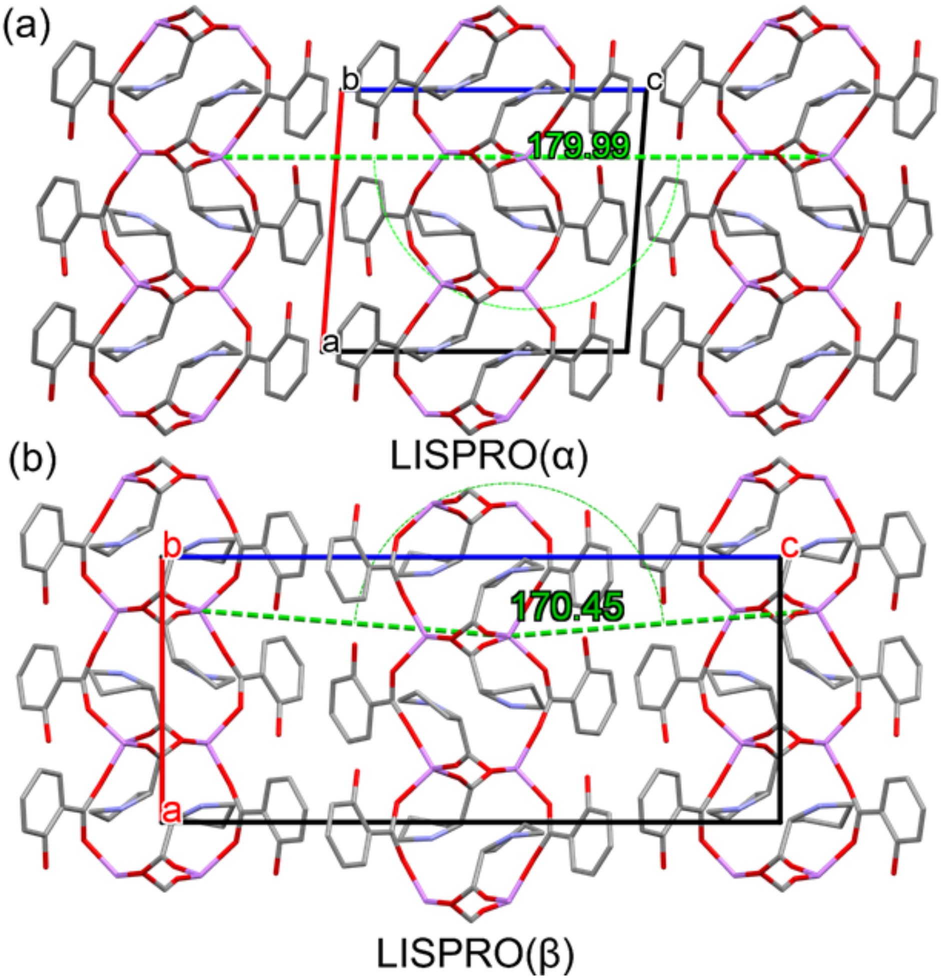

Figure S6: Molecular packing in crystals of (a) LISPRO(α) and (b) LISPRO(β) at 100 K shown along [010] direction. The angles between lithium cations in adjacent square grids are marked in green. For clarity, only major components of the disordered crystal structures are shown and hydrogen atoms have been removed.

L4MPRO(α)

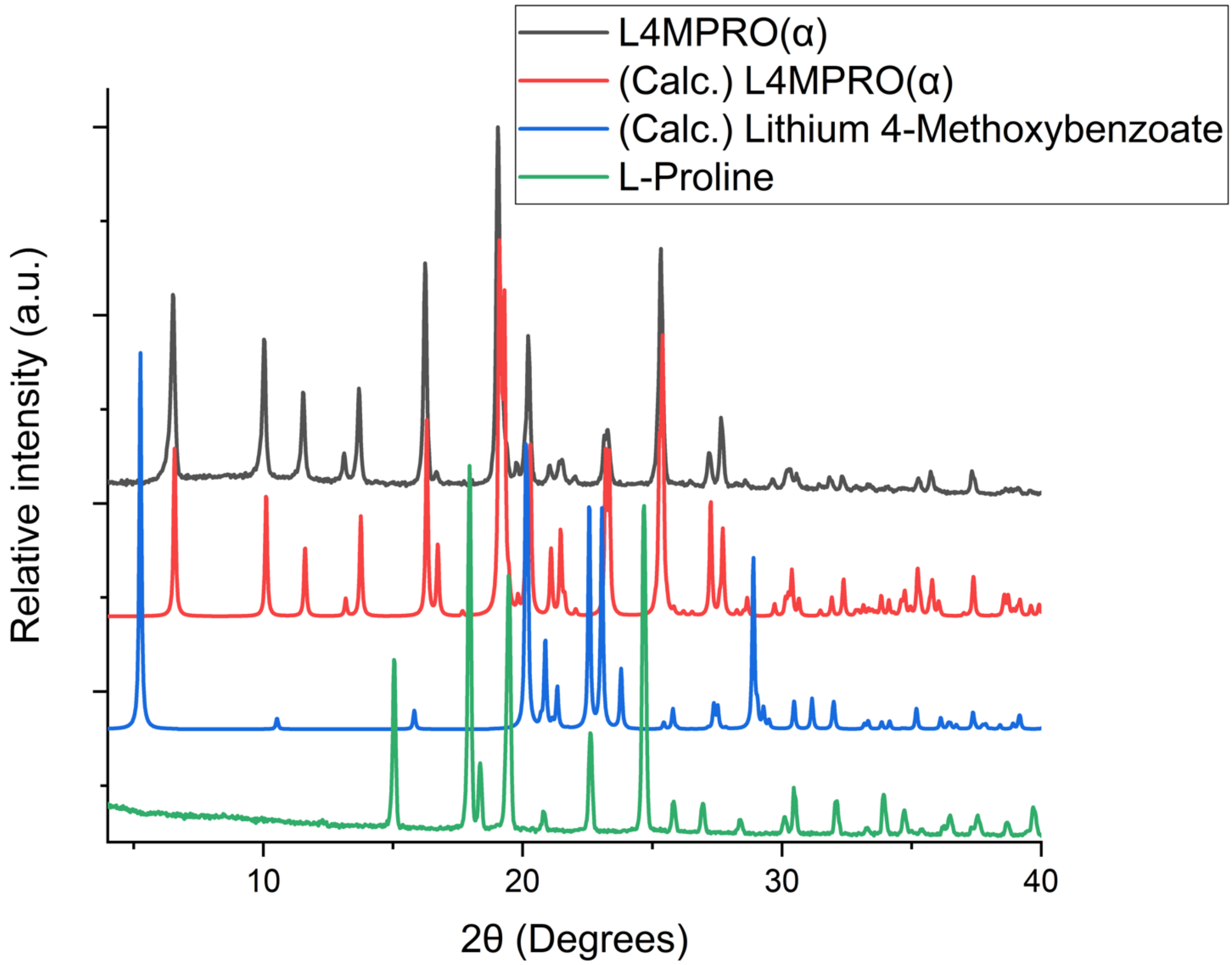

Figure S7: PXRD pattern of experimental L4MPRO(α) (black) and calculated pattern based on a crystal structure determined from SCXRD data at RT (red). Calculated PXRD pattern of lithium 4-methoxybenzoate, experimental PXRD pattern of L-proline, shown in blue and green, respectively, have also been included.

L4MPRO(β)

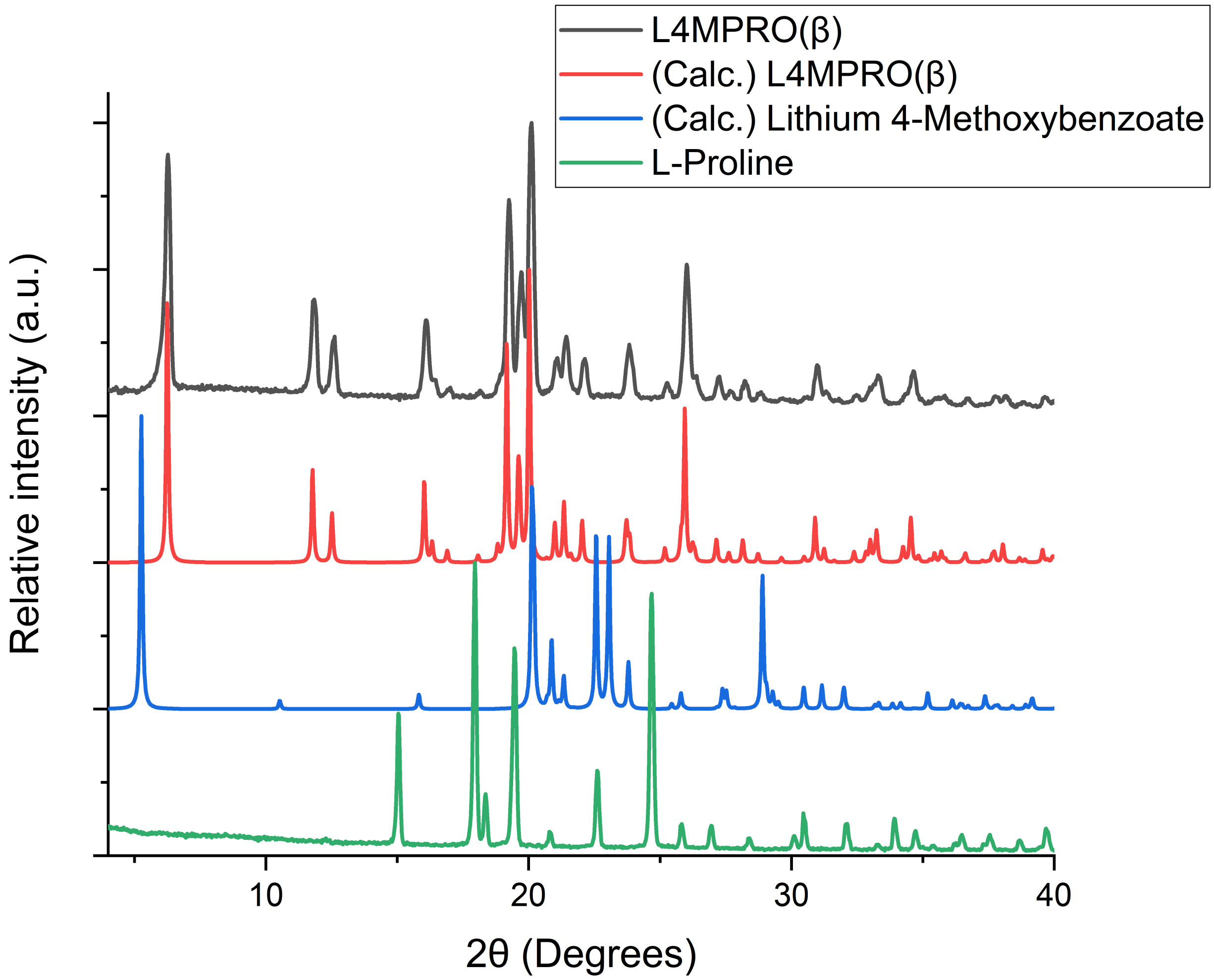

Figure S8: PXRD pattern of experimental L4MPRO(β) (black) and calculated pattern based on a crystal structure determined from SCXRD data at RT (red). Calculated PXRD pattern of lithium 4-methoxybenzoate, and experimental PXRD pattern of L-proline, shown in blue and green, respectively, have been also included.

L4MPRO(γ)

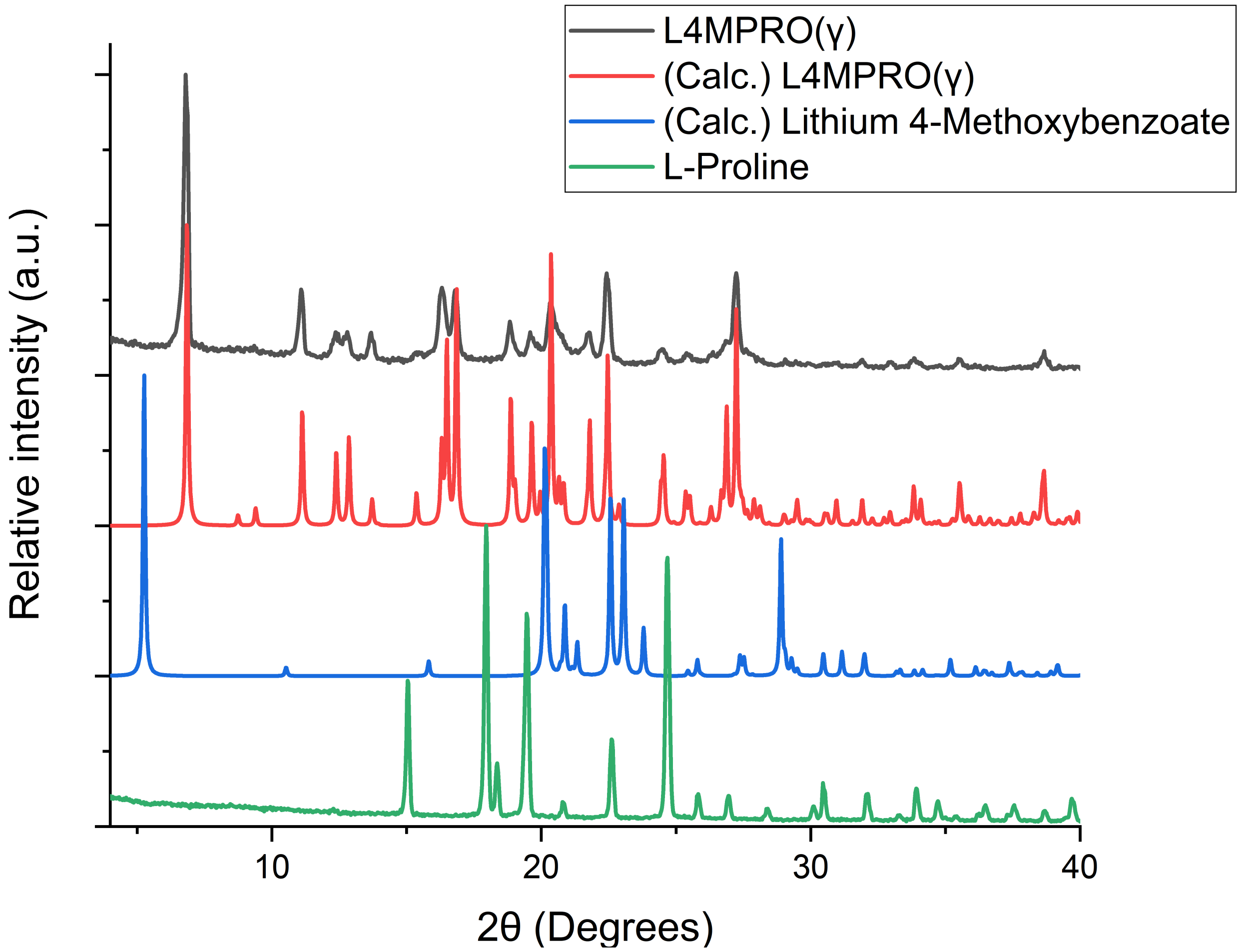

Figure S9: PXRD pattern of experiment L4MPRO(γ) (black) and calculated pattern based on a crystal structure determined from SCXRD data at RT (red). Calculated PXRD pattern of lithium 4-methoxybenzoate, and experimental PXRD pattern of L-proline, shown in blue and green, respectively, have been also included.

L4MPRO Polymorph Comparisons

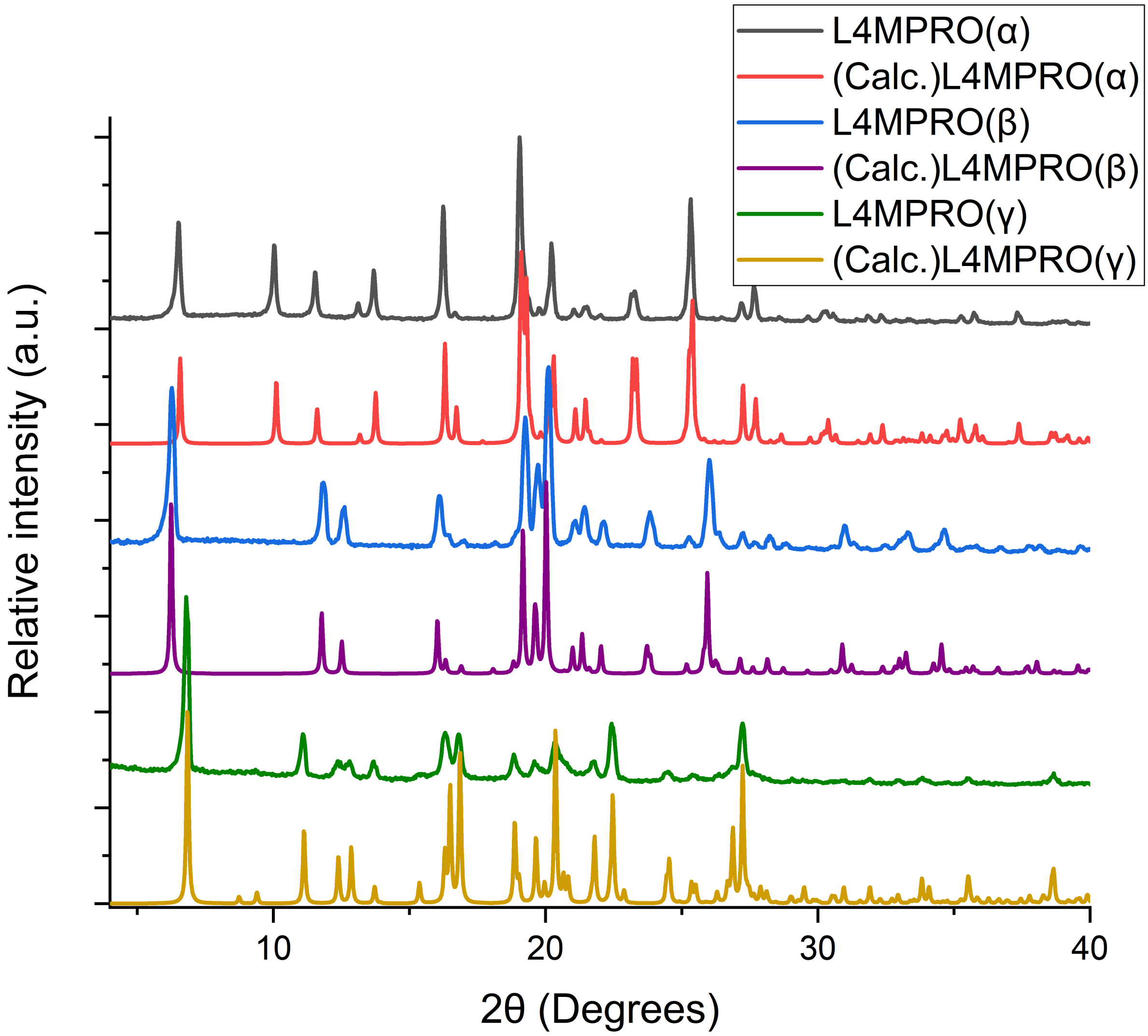

Figure S10: PXRD patterns (calculated and experimental) for all known polymorphs of L4MPRO. The experimental PXRD patterns of polymorphs α, β and γ are shown in black, blue, and green, respectively, while calculated PXRD patterns are in red, purple, and yellow, respectively. All calculated PXRD patterns are based on crystal structures determined from SCXRD data collected at RT.

L4MPRO synthesis by slurry in different solvents

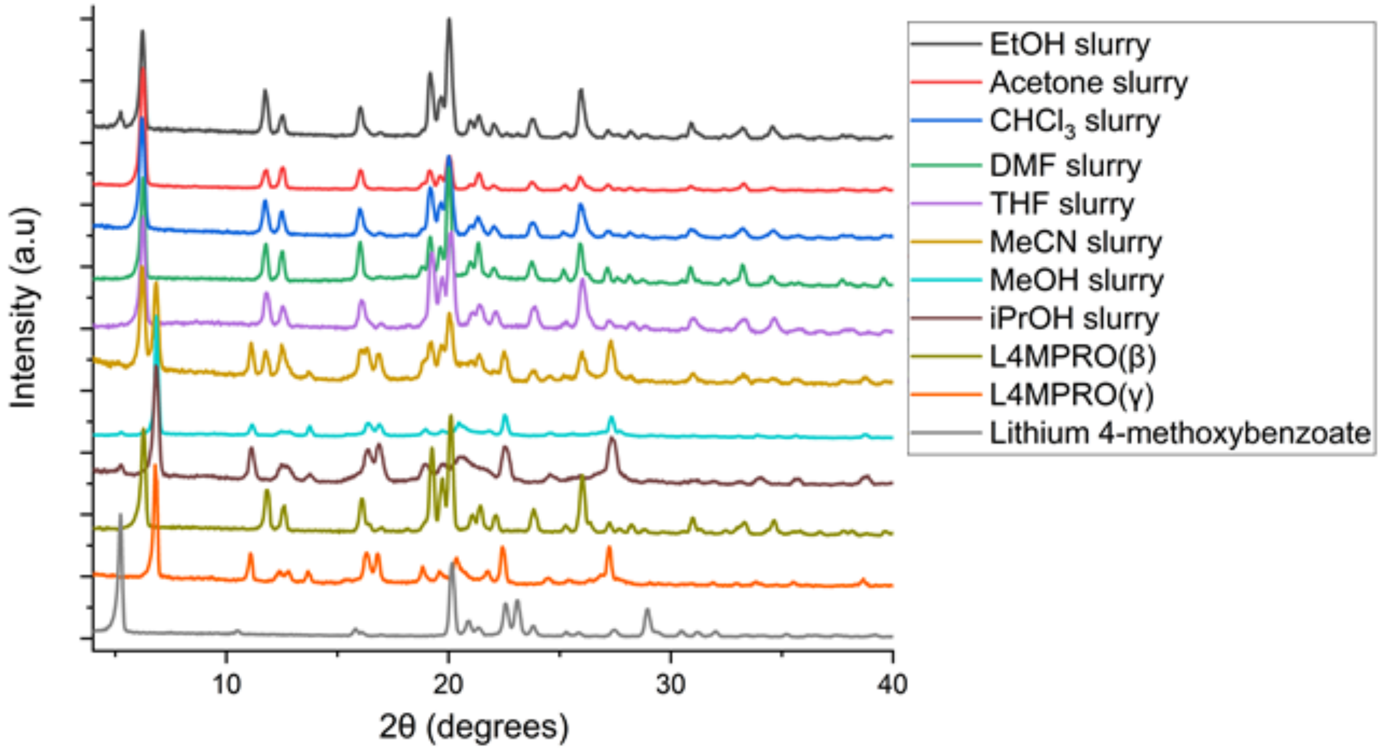

Figure S11: PXRD patterns of the dried powders after lithium 4-methoxybenzoate and L-proline are slurried in different solvents (0.52 mL for MeOH, EtOH, iPrOH, EtOAc, or acetone, 0.17 mL for DMF, $CHCl_3$, THF, or MeCN). PXRD pattern after EtOH slurry is shown in black, after acetone is shown in red, after $CHCl_3$ is shown in dark blue, after DMF is shown in green, after THF is shown in purple, after MeCN is shown in gold, after MeOH shown in light blue, after iPrOH shown in violet. Experimental PXRD patterns of L4MPRO(β), L4MPRO(γ) and lithium 4-methoxybenzoate are shown for comparison with L4MPRO(β) shown in olive, L4MPRO(γ) shown in orange and lithium 4-methoxybenzoate shown in grey. From these results it can be seen that in EtOH, acetone, $CHCl_3$, DMF, and THF L4MPRO(β) forms while in MeOH and iPrOH L4MPRO(γ). The slurry in MeCN displays a mixture of L4MPRO(β) and L4MPRO(γ) patterns. Some patterns, notably the EtOH slurry and iPrOH slurry also contains noticeable quantities of residual lithium 4-methoxybenzoate as seen in the early peak at 5.2°.

L4MPRO crystal structure comparisons

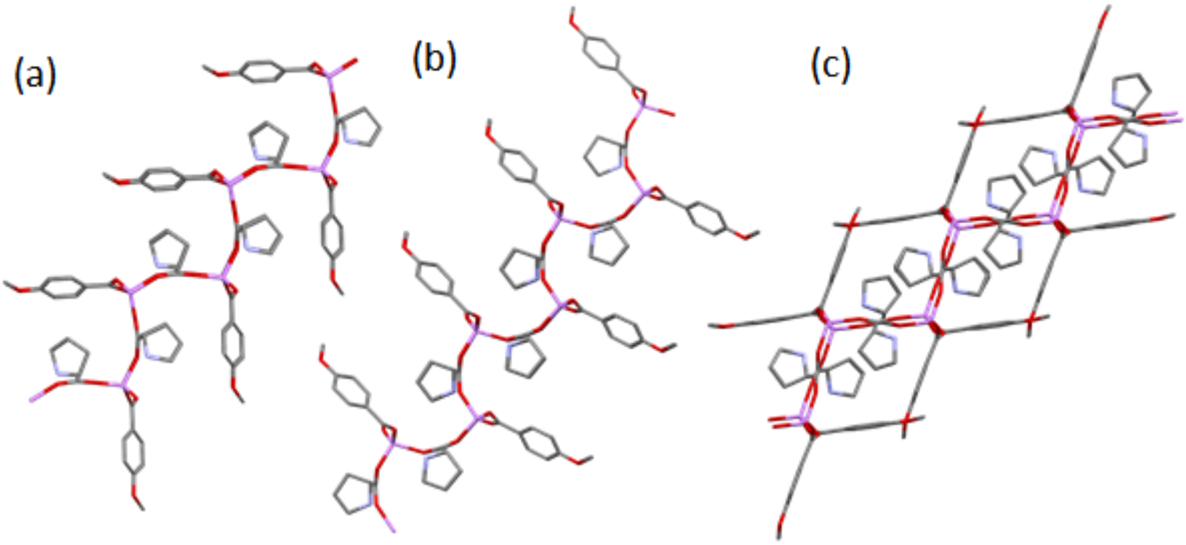

Figure S12: Structure of (a) L4MPRO(α), (b) L4MPRO(β) and (c) L4MPRO(γ) square grid nets (at 100 K), shown along direction [100]. For clarity, hydrogen atoms have been removed.

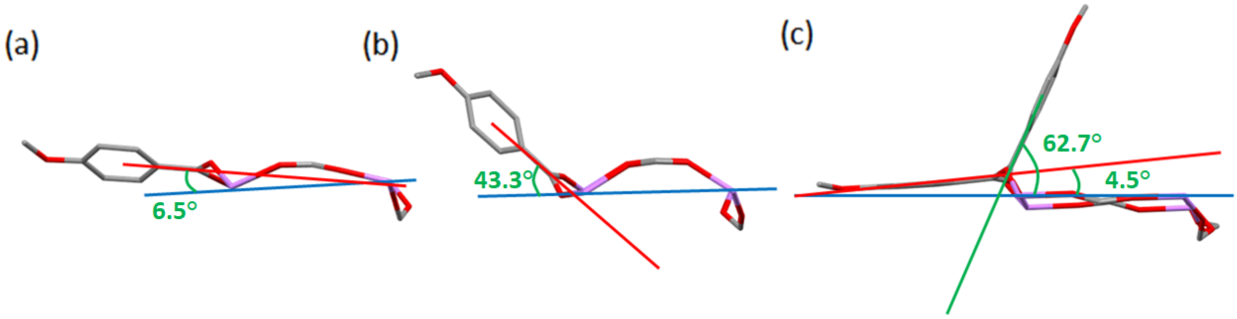

Figure S13: Fragments of square grids in (a) L4MPRO(α), (b) L4MPRO(β) and (c) L4MPRO(γ) nets (at 100 K), shown along direction [100], along with a 4-methoxybenzoate group. To describe the position of the 4-methoxybenzoate, the acute angle (shown as green arcs) between a plane calculated for lithium cations (marked as blue lines) and a plane(s) calculated for the C6-C7 bonds in 4-methoxybenzoate groups (marked as red lines, or an additional green line in L4MPRO(γ)), was measured. The square grid present in L4MPRO(γ) contains two symmetry-independent lithium cations, however each form parallel planes and so measuring from either plane gives the same result. For clarity, L-proline rings and hydrogen atoms have been removed.

Seeding-assisted Mechanochemical Synthesis

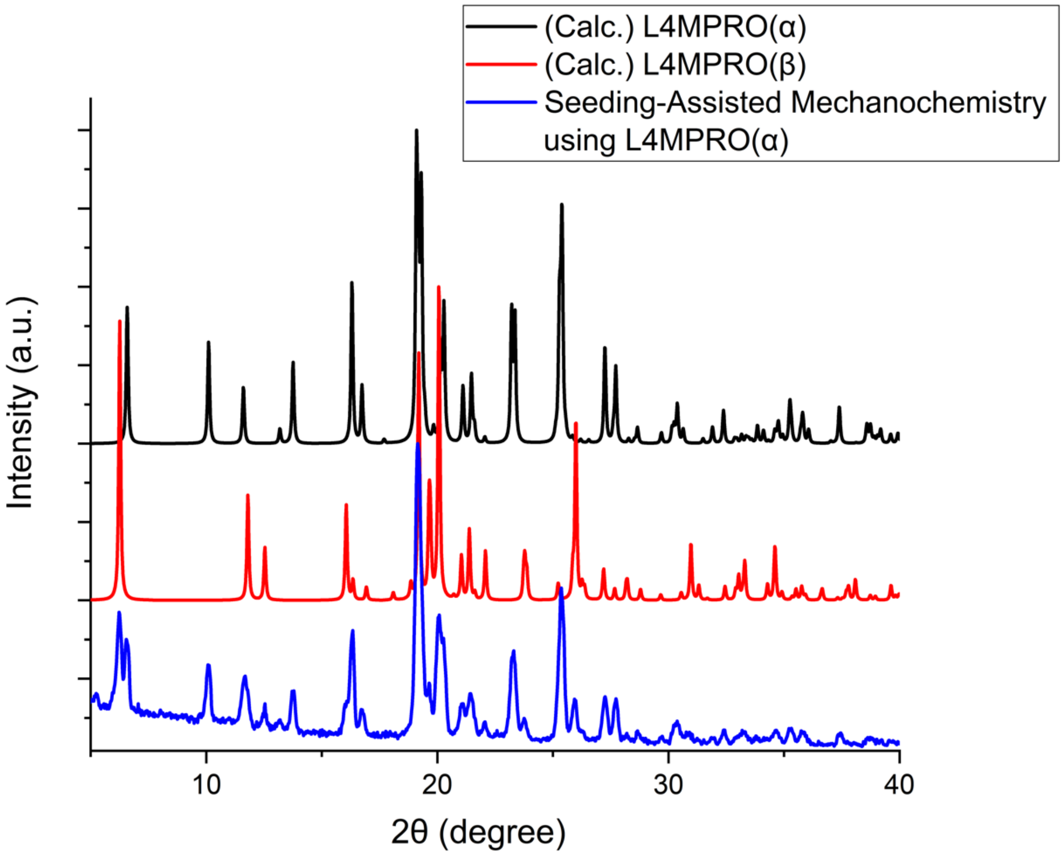

Figure S14: Calculated PXRD patterns of L4MPRO(α) (black) and L4MPRO(β) (red) collected at RT. The experimental PXRD pattern measured for sample obtained from the seeding-assisted grinding of 1:1 lithium 4-methoxybenzoate and L-proline with 5 mol% of L4MPRO(α) and 36 µl of H_2O is shown in blue. Comparison of the relative intensity of the peaks at 6.2° and 6.5° 2θ, shows that mixture of L4MPRO(α) and L4MPRO(β) in approximately 50:50 ratio was received. A small peak visible at 5.2° 2θ can be attributed to residual lithium 4-methoxybenzoate.

FTIR spectroscopy

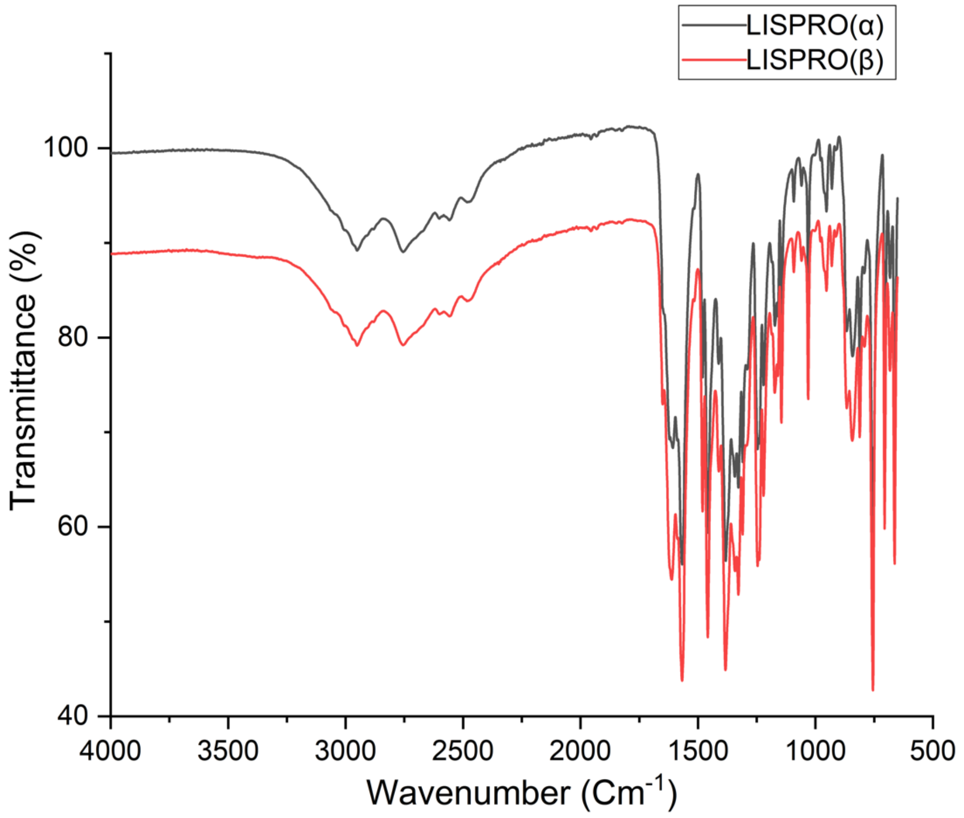

Figure S15: FT-IR comparison of LISPRO(α) and (β). FTIR patterns are near-identical for each polymorph.

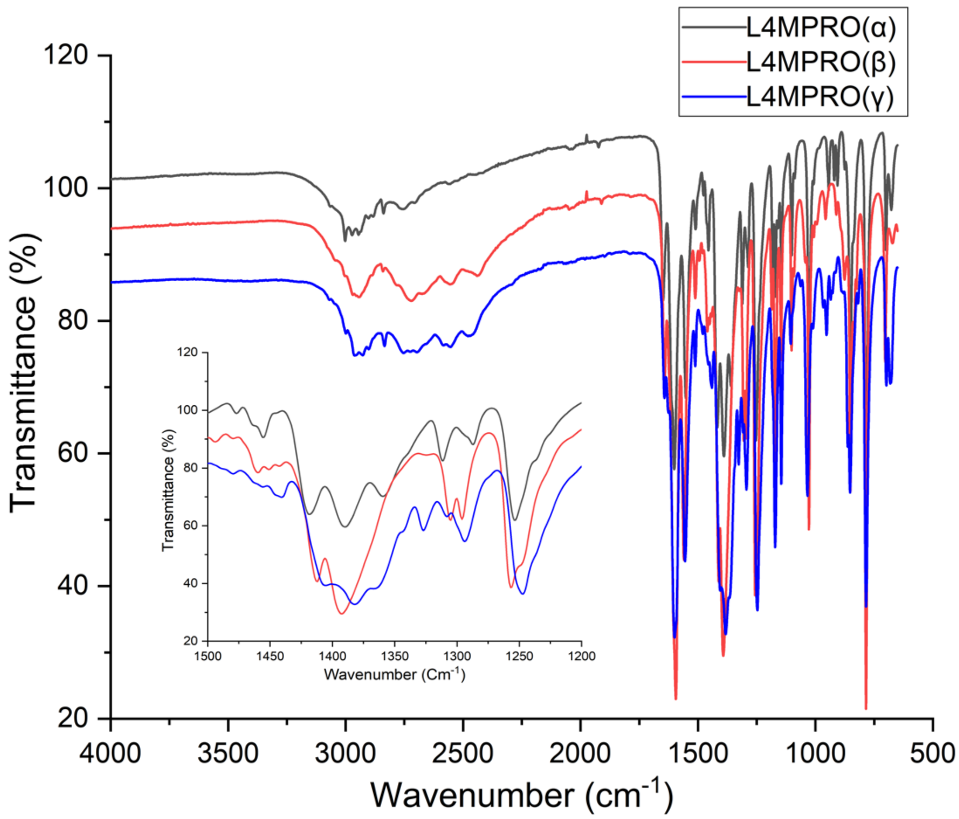

Figure S16: FT-IR comparison of L4MPRO(α), (β) and (γ). The inset graph highlights a key region whereby L4MPRO(α) displays peaks at 1360, 1312 and 1287 cm^{-1}, L4MPRO(β) shows peaks at 1305 and 1297 cm^{-1} and L4MPRO(γ) shows peaks at 1293, 1308, 1327, 1365, 1382 and 1406 cm^{-1}, allowing these polymorphs to be readily distinguishable using IR.

TGA

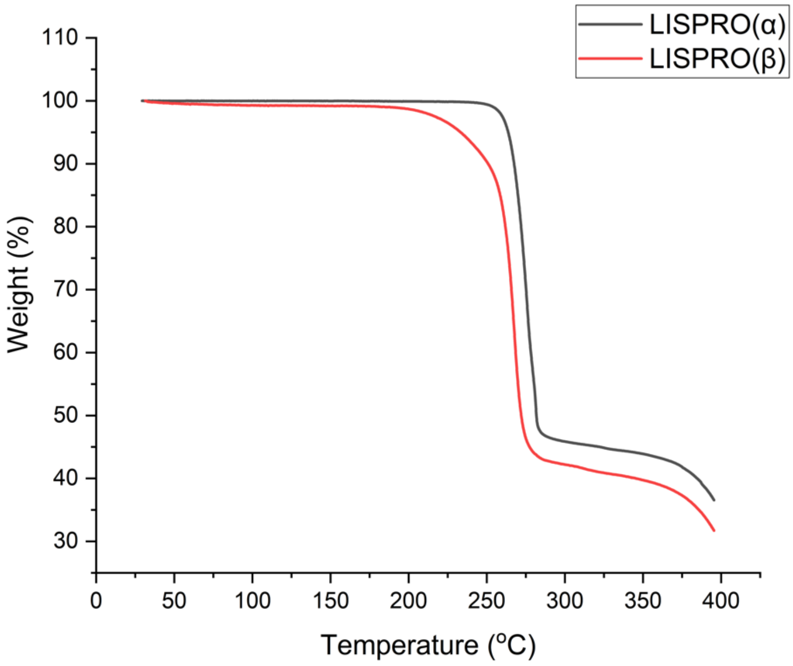

Figure S17: TGA comparison of LISPRO(α) and LISPRO(β). Ramp rate of 10 °C/min from RT to 400 °C.

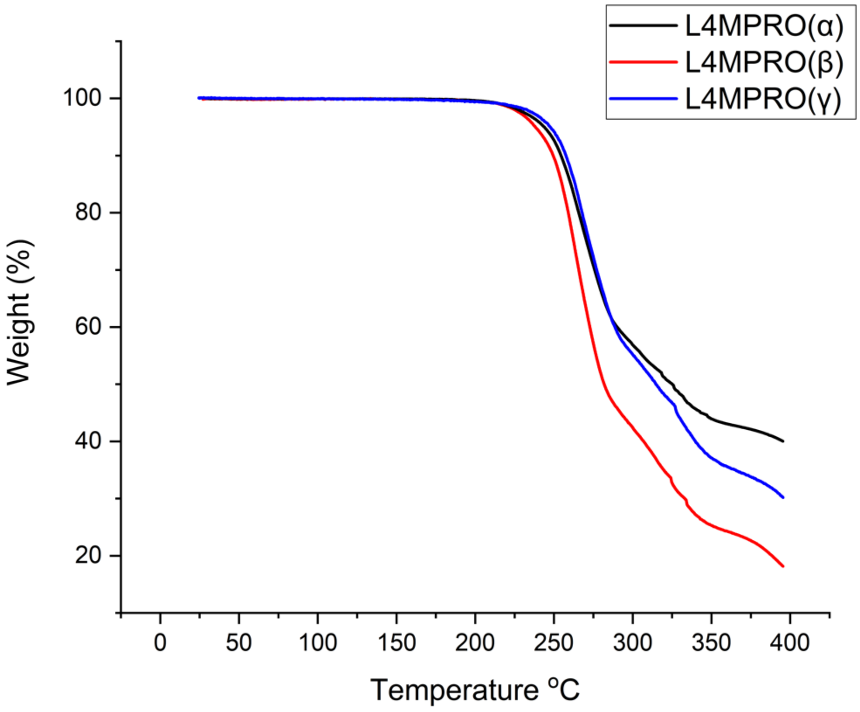

Figure S18: TGA comparison of L4MPRO(α), (β) and (γ). Ramp rate of 10 °C/min from RT to 400 °C.

DSC

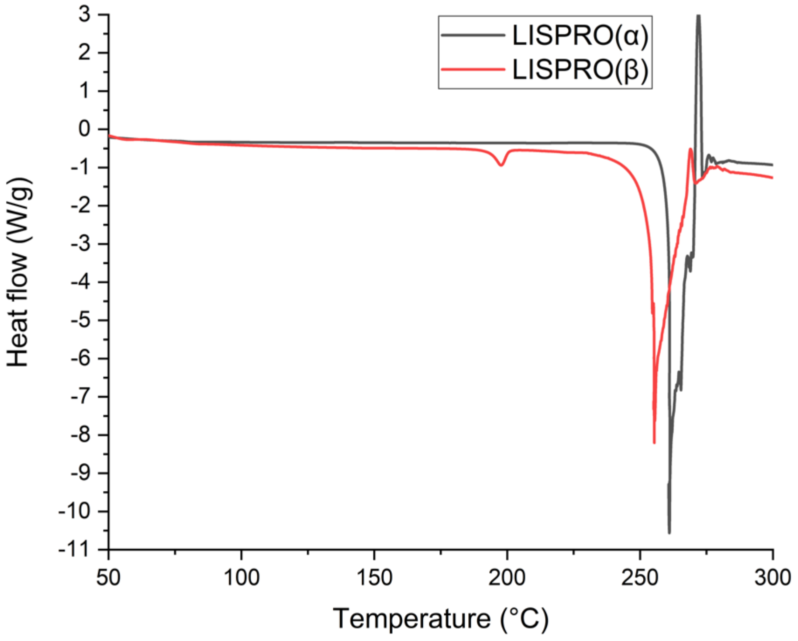

Figure S19: DSC overlay of LISPRO polymorphs. Ramp rate of 10 °C/min from RT to 300 °C. As evidenced in the TGA, thermal degradation of the samples occurs beyond 250 °C, leading to a non-sensical DSC trace. An endothermic event is present for LISPRO(β) at 197.9 °C.

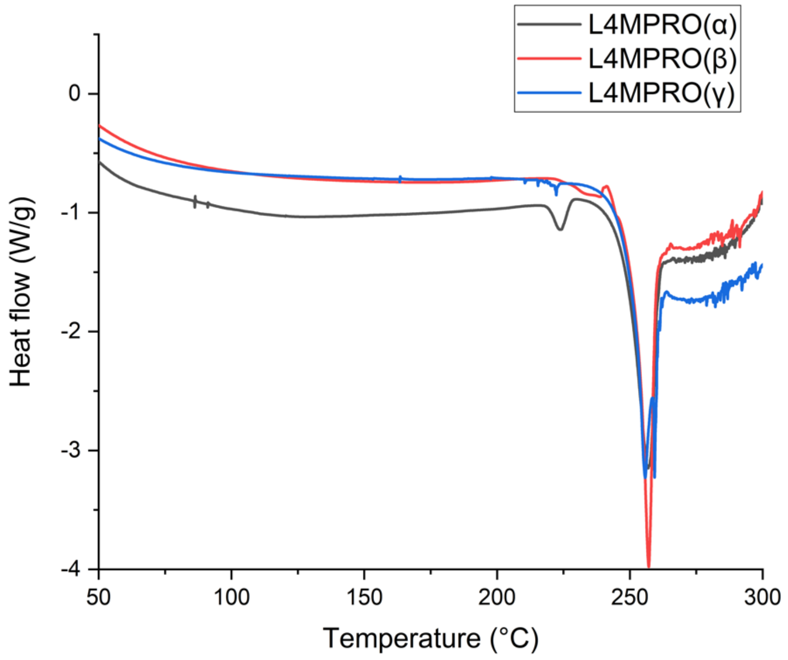

Figure S20: DSC overlay of L4MPRO polymorphs. Ramp rate of 10 °C/min from RT to 300 °C. As evidenced in the TGA, thermal degradation of the samples occurs beyond 250 °C, leading to a non-sensical DSC trace.

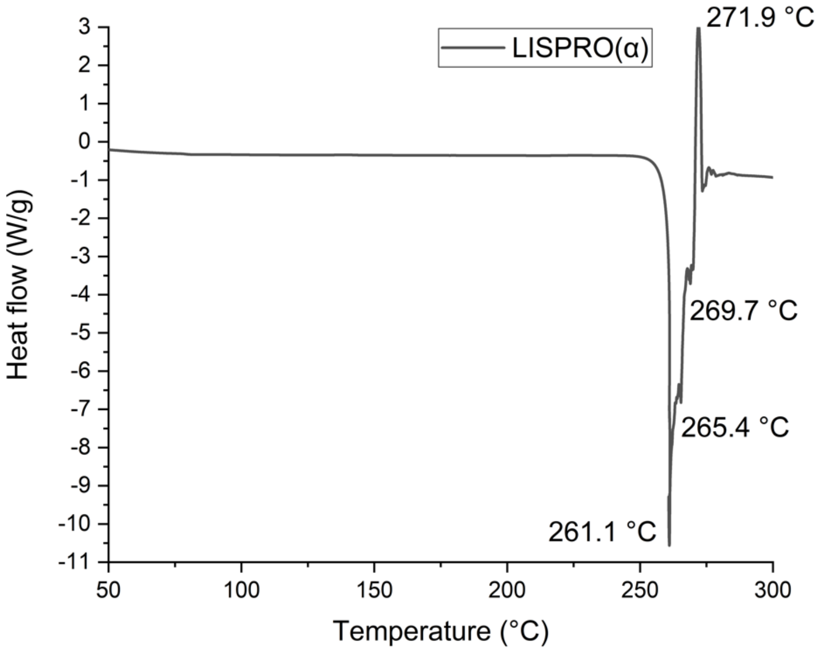

Figure S21: DSC of LISPRO(α). Endothermic thermal events occurred at 261.1 °C, 265.4 °C and 269.7 °C followed by an exothermic even at 271.9 °C.

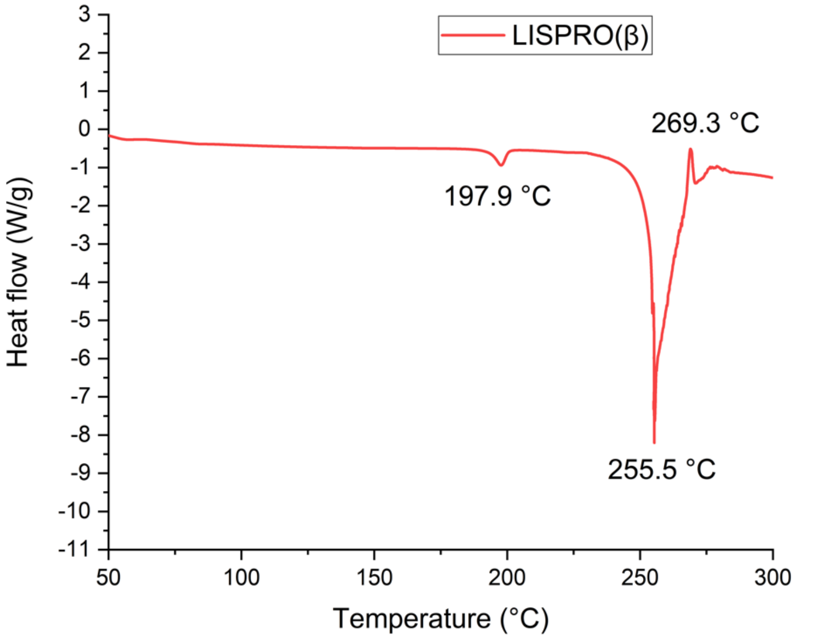

Figure S22: DSC of LISPRO(β). Endothermic thermal events occurred at 197.9 °C and 255.5 °C followed by an exothermic even at 269.3 °C.

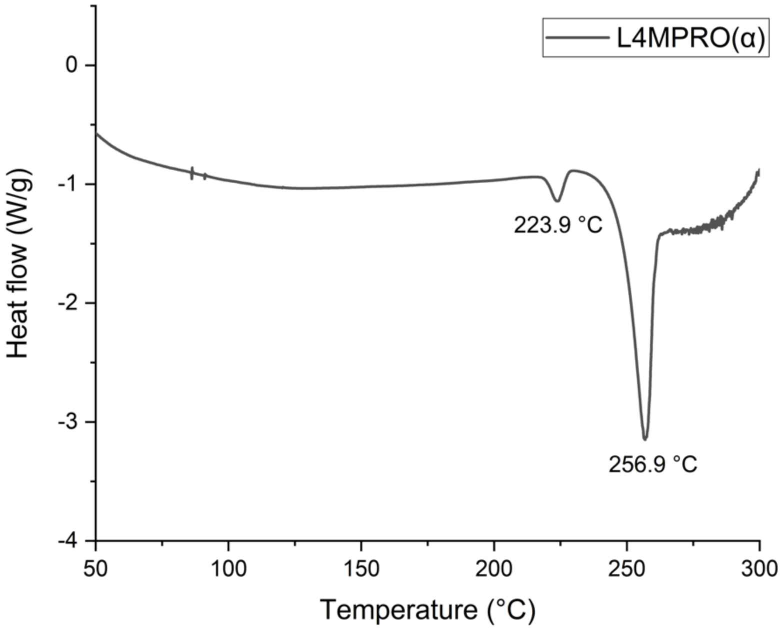

Figure S23: DSC of L4MPRO(α). Endothermic thermal events occurred at 223.9 °C and 256.9 °C.

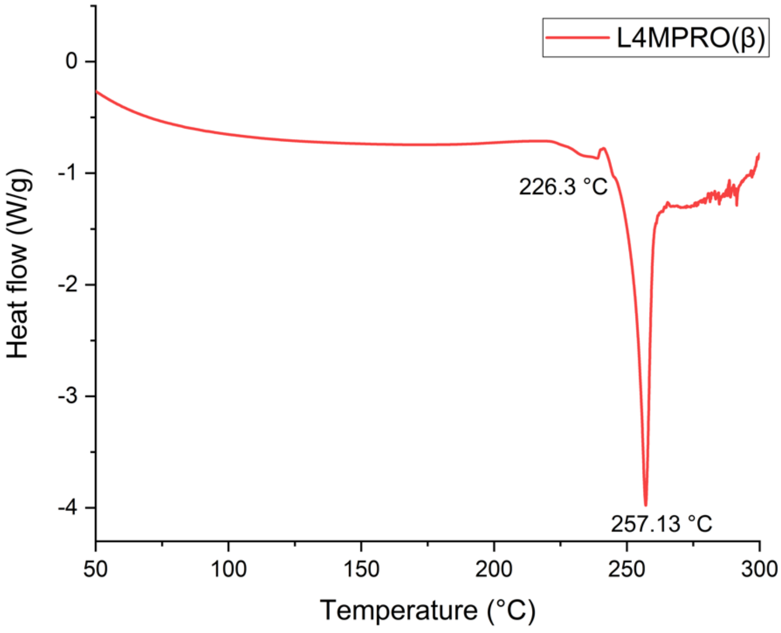

Figure S24: DSC of L4MPRO(β). Endothermic thermal events occurred at 226.3 °C and 257.1 °C.

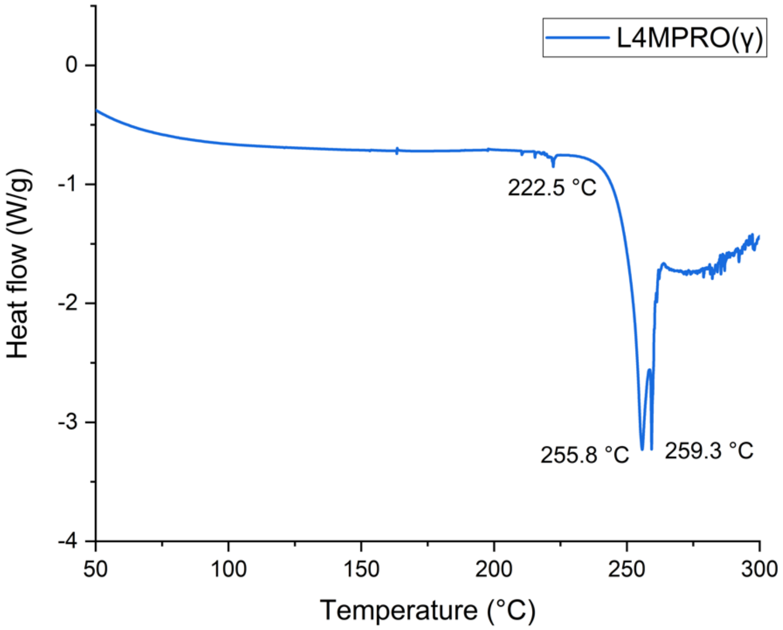

Figure S25: DSC of L4MPRO(β). Endothermic thermal events occurred at 222.5 °C, 255.8 °C and 259.3 °C.

Stability testing

Stability after 6-month ambient storage

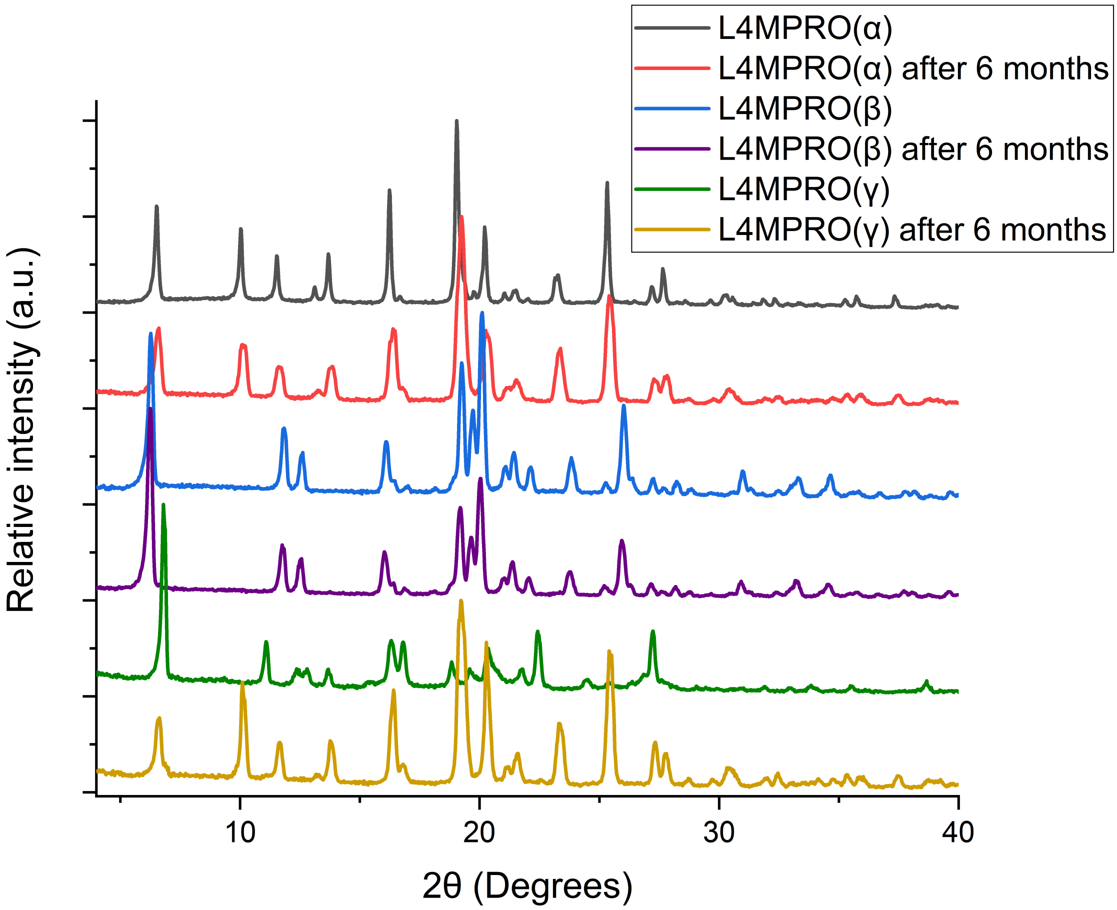

Figure S26: Experimental PXRD patterns collected for as-synthesized samples of L4MPRO polymorphs α, β and γ (shown in black, blue and green, respectively), and samples of α, β and γ polymorphs of L4MPRO after they have been stored under ambient conditions for 6 months (shown in red, purple and yellow, respectively). PXRD patterns for L4MPRO(α) and L4MPRO(β) confirm lack of phase transition, as they remained the same, while changes in the PXRD pattern for L4MPRO(γ) confirms its conversion to L4MPRO(α).

Slurry of LISPRO polymorph mixture

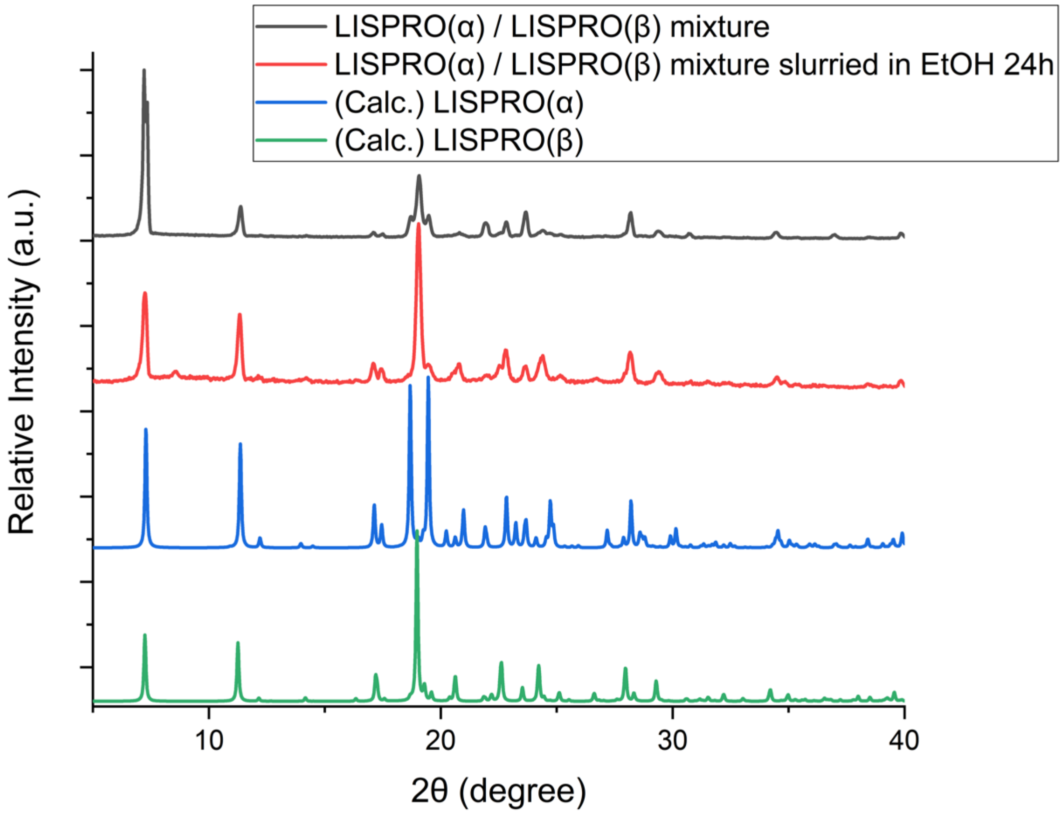

Figure S27: The experimental PXRD patterns collected of a LISPRO polymorph mixture obtained from the rapid evaporation of lithium salicylate and L-proline in water at 70 °C (black), and the same dried sample after slurrying in 1 ml EtOH for 24h (red). The calculated PXRD patterns of LISPRO(α) (blue) and LISPRO(β) (green) collected from RT crystals are shown for comparison. The presence of both polymorphs in the sample from rapid evaporation is evident in the black PXRD from peaks at 7.2°, 18.7° and 19.5° coming from LISPRO(α), and peaks at 7.3° and 19.0° that come from LISPRO(β). From this it can be seen that after slurrying a LISPRO polymorph mixture in EtOH, the sample converts to LISPRO(β).

50:50 Slurry of Polymorph mixtures

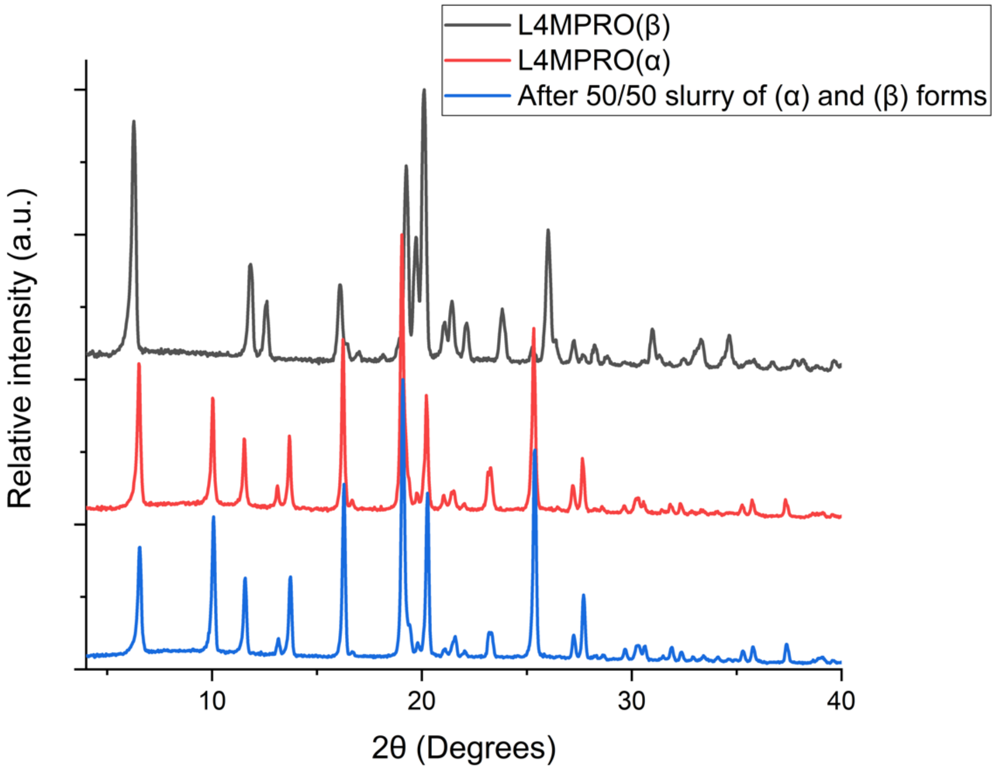

Figure S28: Experimental PXRD patterns of L4MPRO(β) and L4MPRO(α), shown in black and red, respectively, as well as an air-dried sample received after a 50:50 L4MPRO(β):L4MPRO(α) slurry experiment in 400 μl of EtOH for 24 h (blue). A comparison of the PXRD patterns after slurrying shows the resultant powder is pure L4MPRO(α).

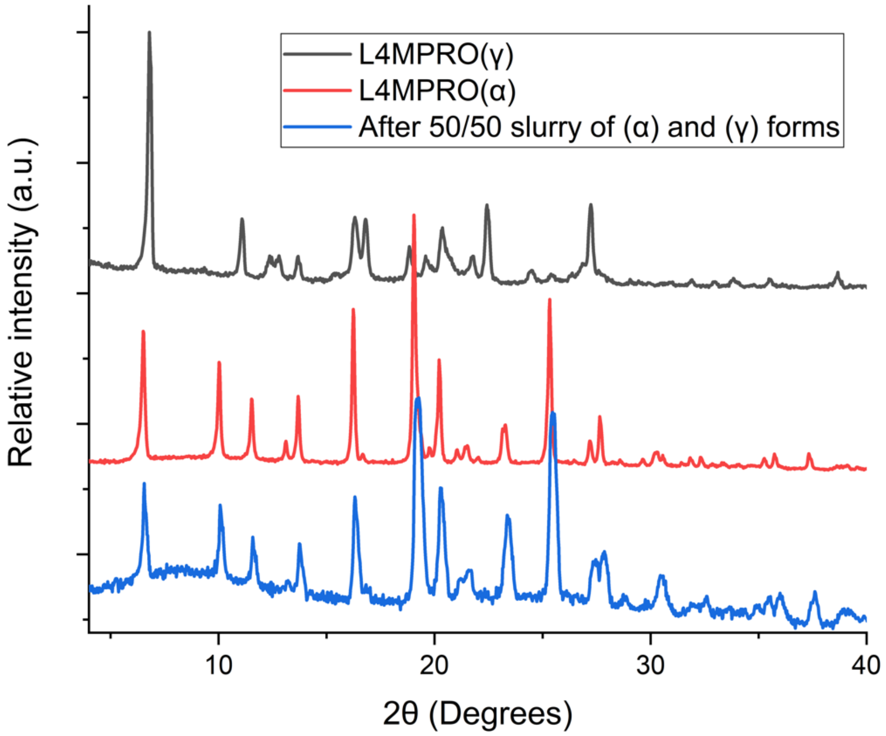

Figure S29: Experimental PXRD patterns of L4MPRO(γ) and L4MPRO(α), shown in black and red, respectively, as well as air-dried sample received as a result of 50:50 L4MPRO(β):L4MPRO(α) slurry experiment in 400 µl of EtOH for 24 h (blue). A comparison of the PXRD patterns after slurrying shows the resultant powder is pure L4MPRO(α).

Stability Testing Under Humidity

LISPRO(β)

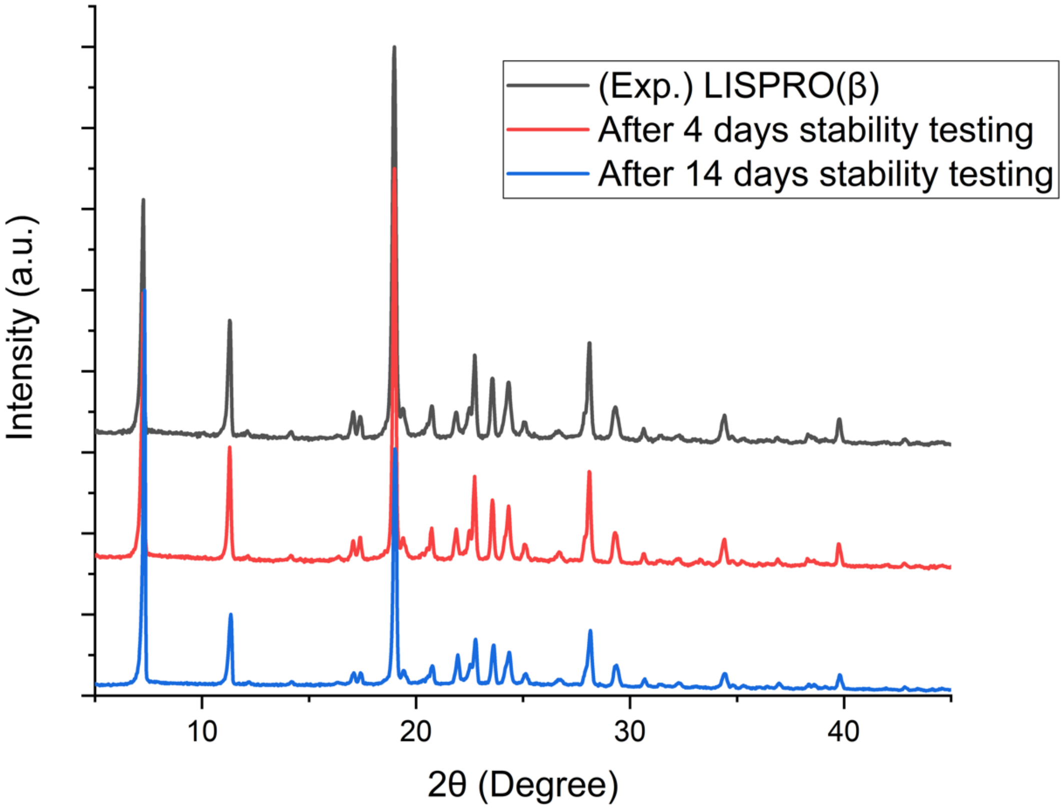

Figure S30: Experimental PXRD patterns of LISPRO(β): as-synthesized (black), after 4 days (red) and 14 days (blue) under accelerated stability testing conditions (75% RH and 40 °C). For accelerated stability experiments, 30 mg of LISPRO(β) were placed in loosely covered glass vials. The PXRD patterns remained unchanged indicating the sample is humidity stable.

L4MPRO(α)

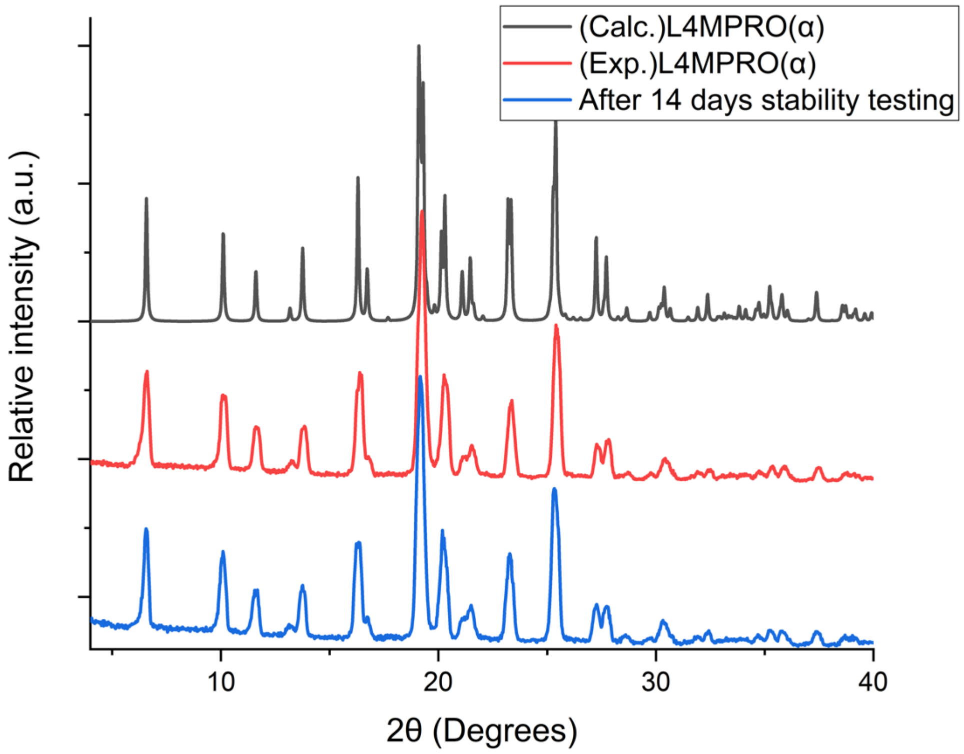

Figure S31: Calculated (from RT-collected structure) and experimental PXRD patterns of L4MPRO(α) shown in black and red, respectively. PXRD pattern measured for L4MPRO(α) sample after 14 days under accelerated stability testing conditions (75% RH and 40 °C) is shown in blue. For accelerated stability experiments 30 mg of LISPRO(α) were placed in loosely covered glass vials. The PXRD pattern remained unchanged indicating the sample is stable under these conditions.

L4MPRO(β)

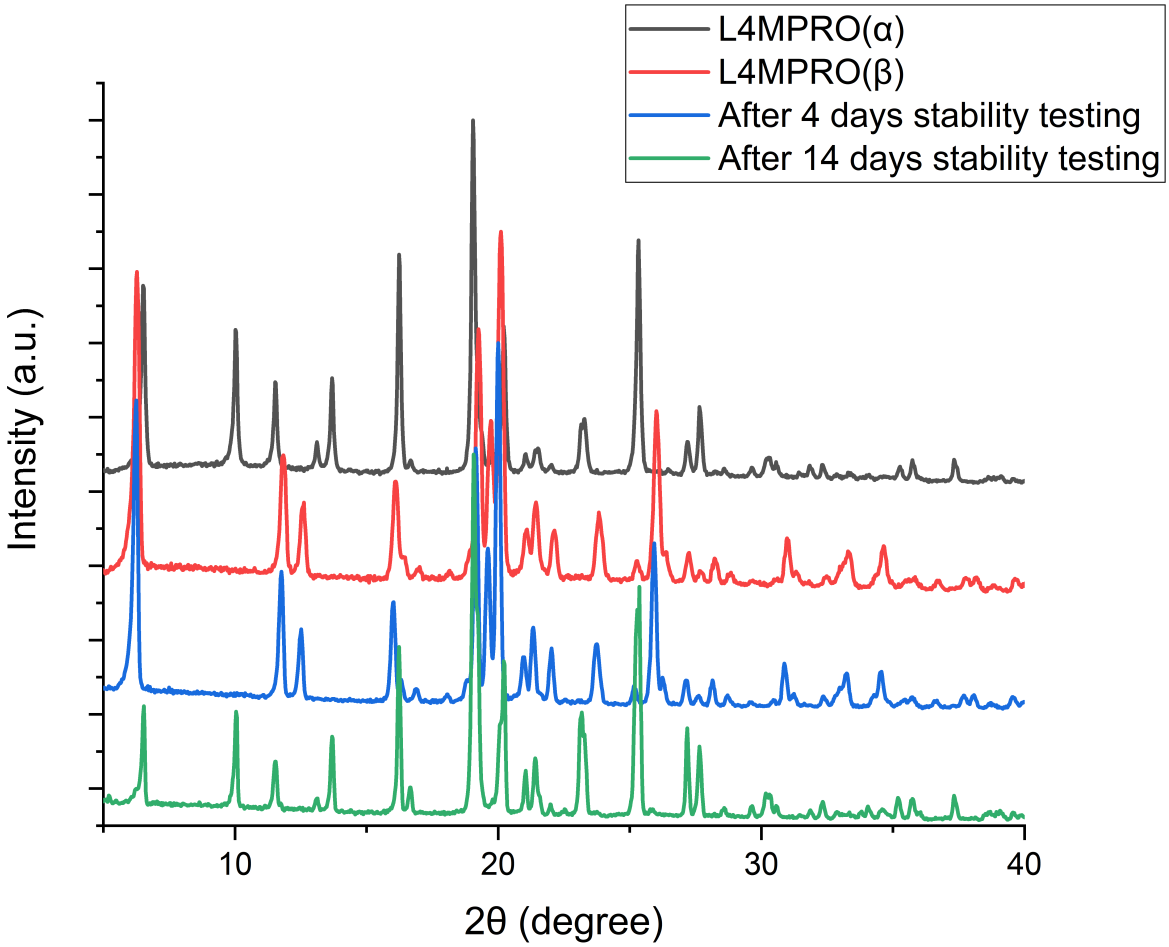

Figure S32: Experimental PXRD patterns for L4MPRO(α) and L4MPRO(β) shown in black and red, respectively. The experimental PXRD patterns for a sample after L4MPRO(β) was exposed to accelerated stability testing conditions (75% RH and 40 °C) for 4 and 14 days, are shown in blue and green, respectively. After 4 days of stability testing, the sample remained unchanged, however after 14 days a near complete polymorphic transformation to L4MPRO(α) was observed, as evidenced by the change in PXRD pattern.

L4MPRO(γ)

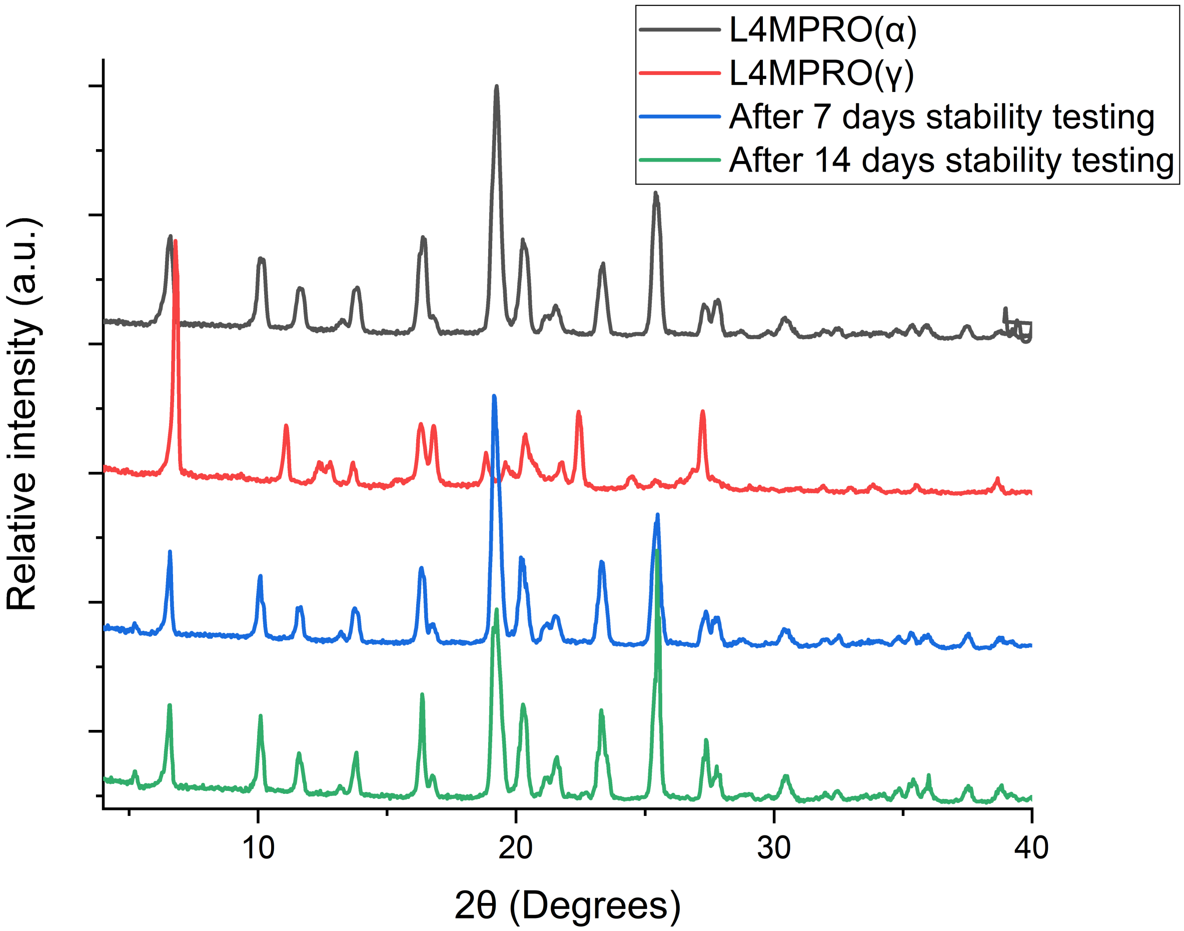

Figure S33: Experimental PXRD patterns for L4MPRO(α) and L4MPRO(γ) are shown in black and red, respectively. Experimental PXRD patterns collected for L4MPRO(γ) sample exposed to accelerated stability testing conditions (75% RH and 40 °C) for 4 and 14 days, are shown in blue and green, respectively. For accelerated stability experiments 30 mg of L4MPRO(γ) were placed in loosely covered glass vials. After 7 days the sample had undergone complete polymorphic transition to the L4MPRO(α) phase and remained in this form for the next 7 days. Some impurity is observed after stability testing, as evidenced by peak at 5.2°, which may be attributed to lithium 4-methoxybenzoate.

Crystallographic details

Table S1. The experimental and crystallographic details for structures of LISPRO polymorphs α and β at 100 K.

	LISPRO(α)	LISPRO(β)
Crystal data		
Chemical formula	$C_{12}H_{14}LiNO_5$	$C_{12}H_{14}LiNO_5$
M_r	259.18	259.18
Crystal system	Monoclinic	Orthorhombic
Space group	$P2_1$	$P2_12_12_1$
Temperature (K)	100	100
a, *b*, *c* (Å)	10.3035 (8), 10.1046 (8), 11.9572 (11)	10.2736 (4), 10.1049 (3), 23.9524 (9)
α, β, γ (°)	90, 94.253 (6), 90	90, 90, 90
V (Å^3)	1241.47 (18)	2486.59 (15)
ρ_{calc} (g·cm^{-3})	1.387	1.385
Z/Z'	4/2	8/2
Radiation type	Cu *K*α	Cu *K*α
μ (mm^{-1})	0.90	0.90
Crystal size (mm)	0.43 × 0.24 × 0.10	0.27 × 0.14 × 0.07
Data collection		
Absorption correction	Numerical	Multi-scan
T_{min}, T_{max}	0.536, 0.752	0.566, 0.737
No. of measured, independent and observed [$I > 2\sigma(I)$] reflections	9441, 3896, 3541	42030, 4743, 4383
R_{int}	0.057	0.076
$(\sin \theta/\lambda)_{max}$ (Å^{-1})	0.582	0.610
Refinement		
$R[F^2 > 2\sigma(F^2)]$, $wR(F^2)$ $[F^2 > 2\sigma(F^2)]$, S	0.075, 0.197, 1.05	0.050, 0.125, 1.12
R, $wR(F^2)$ (all data)	0.083, 0.205	0.054, 0.129
No. of reflections	3896	4743
No. of parameters	422	434
No. of restraints	200	55
H-atom treatment	H-atom parameters constrained	H-atom parameters constrained
$\Delta\rho_{max}$, $\Delta\rho_{min}$ (e Å^{-3})	0.49, −0.37	0.34, −0.28
Absolute structure	Flack x determined using 1454 quotients [(I+)-(I-)]/[(I+)+(I-)][a]	Flack x determined using 1787 quotients [(I+)-(I-)]/[(I+)+(I-)][a]
Absolute structure parameter	0.5 (2)[b]	−0.03 (8)[b]

[a] (Parsons, Flack and Wagner, Acta Cryst. B69 (2013) 249-259).; [b] As the studied compound are weak anomalous scatterers (do not include atoms heavier than Si) the absolute structure parameters are meaningless and hence were removed from the CIFs. The absolute structure was determined based on the known absolute configuration of L-proline.

Table S2. The experimental and crystallographic details for structures of LISPRO polymorphs α and β at 298 K.

	LISPRO(α)	LISPRO(β)
Crystal data		
Chemical formula	$C_{12}H_{14}LiNO_5$	$C_{12}H_{14}LiNO_5$
M_r	259.18	259.18
Crystal system	Monoclinic	Orthorhombic
Space group	$P2_1$	$P2_12_12_1$
Temperature (K)	298	298
a, *b*, *c* (Å)	10.3602 (9), 10.1537 (9), 12.1414 (12)	10.308 (2), 10.268 (2), 24.386 (5)
α, β, γ (°)	90, 93.356 (5), 90	90, 90, 90
V (Å^3)	1275.0 (2)	2581.1 (9)
ρ_{calc} (g·cm^{-3})	1.350	1.334
Z/Z'	4/2	8/2
Radiation type	Cu *K*α	Cu *K*α
μ (mm^{-1})	0.87	0.86
Crystal size (mm)	0.32 × 0.18 × 0.04	0.31 × 0.29 × 0.06
Data collection		
Absorption correction	Multi-scan	Multi-scan
T_{min}, T_{max}	0.511, 0.734	0.549, 0.752
No. of measured, independent and observed [$I > 2\sigma(I)$] reflections	19130, 3877, 3075	20543, 3822, 3309
R_{int}	0.081	0.054
$(\sin \theta/\lambda)_{max}$ (Å^{-1})	0.567	0.569
Refinement		
$R[F^2 > 2\sigma(F^2)]$, $wR(F^2)$ [$F^2 > 2\sigma(F^2)$], S	0.097, 0.260, 1.06	0.048, 0.131, 1.10
R, $wR(F^2)$ (all data)	0.113, 0.282	0.058, 0.144
No. of reflections	3877	3822
No. of parameters	432	435
No. of restraints	224	98
H-atom treatment	H-atom parameters constrained	H-atom parameters constrained
$\Delta\rho_{max}$, $\Delta\rho_{min}$ (e Å^{-3})	0.52, −0.34	0.33, −0.18
Absolute structure	Flack x determined using 1176 quotients [(I+)-(I-)]/[(I+)+(I-)] [a]	Flack x determined using 1298 quotients [(I+)-(I-)]/[(I+)+(I-)] [a]
Absolute structure parameter	0.2 (2) [b]	0.15 (9) [b]

[a] (Parsons, Flack and Wagner, Acta Cryst. B69 (2013) 249-259); [b] As the studied compounds are weak anomalous scatterers (they do not include atoms heavier than Si) the absolute structure parameters are unreliable and hence were removed from the CIFs. The absolute structure was determined based on the known absolute configuration of L-proline.

Table S3. The experimental and crystallographic details for structures of L4MPRO polymorphs α, β and γ at 100 K.

	L4MPRO(α)	L4MPRO(β)	L4MPRO(γ)
Crystal data			
Chemical formula	$C_{13}H_{16}LiNO_5$	$C_{13}H_{16}LiNO_5$	$C_{13}H_{16}LiNO_5$
M_r	273.21	273.21	273.21
Crystal system	Orthorhombic	Monoclinic	Monoclinic
Space group	$P2_12_12_1$	$P2_1$	$P2_1$
Temperature (K)	100	100	100
a, *b*, *c* (Å)	5.3718 (1), 9.1760 (3), 26.5432 (7)	5.4154 (2), 8.7562 (4), 14.1554 (7)	10.4879 (13), 10.0527 (12), 13.2148 (16)
α, β, γ (°)	90, 90, 90	90, 95.493 (2), 90	90, 107.653 (4), 90
V (Å^3)	1308.36 (6)	668.14 (5)	1327.7 (3)
ρ_{calc} (g·cm^{-3})	1.387	1.358	1.367
Z/Z'	4/1	2/1	4/2
Radiation type	Mo *K*α	Mo *K*α	Mo *K*α
μ (mm^{-1})	0.11	0.10	0.10
Crystal size (mm)	0.48 × 0.09 × 0.04	0.30 × 0.22 × 0.02	0.28 × 0.19 × 0.07
Data collection			
Absorption correction	Multi-scan	Multi-scan	Numerical
T_{min}, T_{max}	0.685, 0.746	0.658, 0.746	0.648, 0.746
No. of measured, independent and observed [$I > 2\sigma(I)$] reflections	17511, 3674, 3291	15662, 3312, 2947	26994, 6669, 5272
R_{int}	0.034	0.038	0.058
$(\sin \theta/\lambda)_{max}$ (Å^{-1})	0.695	0.667	0.671
Refinement			
$R[F^2 > 2\sigma(F^2)]$, $wR(F^2)$ $[F^2 > 2\sigma(F^2)]$, S	0.034, 0.075, 1.04	0.034, 0.077, 1.04	0.064, 0.156, 1.05
R, $wR(F^2)$ (all data)	0.042, 0.079	0.041, 0.080	0.089, 0.168
No. of reflections	3674	3312	6669
No. of parameters	182	182	359
No. of restraints	0	1	28
H-atom treatment	H-atom parameters constrained		
$\Delta\rho_{max}$, $\Delta\rho_{min}$ (e Å^{-3})	0.29, −0.17	0.25, −0.21	0.72, −0.36
Absolute structure	Flack x determined using 1235 quotients [(I+)-(I-)]/[(I+)+(I-)][a].	Flack x determined using 1260 quotients [(I+)-(I-)]/[(I+)+(I-)]. [a]	Flack x determined using 2042 quotients [(I+)-(I-)]/[(I+)+(I-)]. [a]
Absolute structure parameter	−0.1 (3) [b]	−0.4 (3) [b]	0.8 (5) [b]

[a] (Parsons, Flack and Wagner, Acta Cryst. B69 (2013) 249-259); [b] As the studied compounds are weak anomalous scatterers (they do not include atoms heavier than Si) the absolute structure parameters are unreliable and were removed from the CIFs. The absolute structure was determined based on the known absolute configuration of L-proline. Parsons, Flack and Wagner, Acta Cryst. B69 (2013) 249-259).

Table S4. The experimental and crystallographic details for structures of L4MPRO polymorphs α, β and γ at RT.

	L4MPRO(α)	L4MPRO(β)	L4MPRO(γ)
Crystal data			
Chemical formula	$C_{13}H_{16}LiNO_5$	$C_{13}H_{16}LiNO_5$	$C_{13}H_{16}LiNO_5$
M_r	273.21	273.21	273.21
Crystal system	Orthorhombic	Monoclinic	Monoclinic
Space group	$P2_12_12_1$	$P2_1$	$P2_1$
Temperature (K)	293	298	298
a, *b*, *c* (Å)	5.4014 (5), 9.2466 (10), 26.852 (3)	5.4513 (2), 8.8619 (4), 14.2030 (6)	10.5677 (4), 10.0854 (5), 13.4875 (7)
α, β, γ (°)	90, 90, 90	90, 95.698 (3), 90	90, 107.030 (3), 90
V (Å^3)	1341.1 (2)	682.74 (5)	1374.46 (11)
ρ_{calc} (g·cm^{-3})	1.353	1.329	1.320
Z/Z'	4/1	2/1	4/2
Radiation type	Cu *K*α	Cu *K*α	Cu *K*α
μ (mm^{-1})	0.86	0.84	0.84
Crystal size (mm)	0.32 × 0.03 × 0.02	0.43 × 0.18 × 0.08	0.29 × 0.05 × 0.05
Data collection			
Absorption correction	Multi-scan	Multi-scan	Multi-scan
T_{min}, T_{max}	0.575, 0.753	0.313, 0.753	0.603, 0.753
No. of measured, independent and observed [$I > 2\sigma(I)$] reflections	12195, 2280, 1725	5694, 2205, 1734	4558, 4558, 3752
R_{int}	0.107	0.076	0.075
$(\sin \theta/\lambda)_{max}$ (Å^{-1})	0.588	0.596	0.595
Refinement			
$R[F^2 > 2\sigma(F^2)]$, $wR(F^2)$ $[F^2 > 2\sigma(F^2)]$, S	0.055, 0.137, 1.02	0.077, 0.187, 1.05	0.055, 0.123, 1.11
R, $wR(F^2)$ (all data)	0.076, 0.154	0.096, 0.215	0.074, 0.134
No. of reflections	2280	2205	4558
No. of parameters	183	192	360
No. of restraints	0	1	1
H-atom treatment	H-atom parameters constrained		
$\Delta\rho_{max}$, $\Delta\rho_{min}$ (e Å^{-3})	0.22, −0.24	0.33, −0.46	0.18, −0.25
Absolute structure	Flack x determined using 573 quotients [(I+)-(I-)]/[(I+)+(I-)][a].	Flack x determined using 598 quotients [(I+)-(I-)]/[(I+)+(I-)]. [a]	Flack x determined using 1407 quotients [(I+)-(I-)]/[(I+)+(I-)] [a]
Absolute structure parameter	−0.1 (3) [b]	0.3 (3) [b]	0.24 (18) [b]

[a] (Parsons, Flack and Wagner, Acta Cryst. B69 (2013) 249-259).; [b] As the studied compound are weak anomalous scatterers (do not include atoms heavier than Si) the absolute structure parameters are meaningless and hence were removed from the CIFs. The absolute structure was determined based on the known absolute configuration of L-proline.

Table S5. Selected geometric parameters for Li—O bonds in square grid network of LISPRO(β) at 100 K.

The Li—O bonds			
Li—O bond	*d* (Å)	Li—O bond	*d* (Å)
Li1—O1	1.903 (7)	Li1A—O1A	1.897 (7)
Li1—O2A^{iii}	1.913 (7)	Li1A—O2^{v}	1.903 (7)
Li1—O3	1.856 (9)	Li1A—O3A	1.887 (7)
Li1—O4A^{iv}	1.897 (7)	O4—Li1A	1.919 (9)
Li1—O4′	1.90 (4)	Li1A—O3′	1.92 (4)
The O—Li—O angles			
O—Li—O angle	∠ (°)	O—Li—O angle	∠ (°)
O1A—Li1A—O2^{v}	119.3 (4)	O1—Li1—O2A^{iii}	117.4 (3)
O1A—Li1A—O3'	100.0 (15)	O3—Li1—O1	108.3 (4)
O1A—Li1A—O4	106.1 (4)	O3—Li1—O2A^{iii}	105.8 (4)
O2^{v}—Li1A—O3'	106.4 (15)	O3—Li1—O4A^{iv}	107.8 (4)
O2^{v}—Li1A—O4	111.1 (4)	O4A^{iv}—Li1—O1	111.6 (3)
O3A—Li1A—O1A	113.9 (3)	O4A^{iv}—Li1—O2A^{iii}	105.3 (3)
O3A—Li1A—O2^{v}	102.3 (3)	O4A^{iv}—Li1—O4'	95.4 (9)
O3A—Li1A—O3'	115.4 (10)	O4'—Li1—O1	110.0 (14)
O3A—Li1A—O4	103.1 (4)		
The C—O—Li—O torsion angles			
C—O—Li—O torsion angle	∠ (°)	C—O—Li—O torsion angle	∠ (°)
C1A—O1A—Li1A—O2^{v}	141.6 (14)	C6A—O3A—Li1A—O3'	−84 (2)
C1A—O1A—Li1A—O3A	−97.4 (15)	C6A—O3A—Li1A—O4	−83.7 (11)
C1A—O1A—Li1A—O3'	26 (2)	C6—O3—Li1—O1	−13.1 (16)
C1A—O1A—Li1A—O4	15.3 (17)	C6—O3—Li1—O2A^{iii}	−139.8 (14)
C6A—O3A—Li1A—O1A	30.8 (12)	C6—O3—Li1—O4A^{iv}	107.8 (15)
C6A—O3A—Li1A—O2^{v}	160.9 (9)		

Symmetry code(s): (iii) *x*+1/2, −*y*+1/2, −*z*+1; (iv) *x*+1, *y*, *z*; (v) *x*−1/2, −*y*+3/2, −*z*+1.

Table S6. Selected geometric parameters for Li—O bonds in square grid network of LISPRO(β) at 298 K.

The Li—O bonds			
Li—O bond	*d* (Å)	Li—O bond	*d* (Å)
Li1—O1	1.930 (7)	Li1A—O1A	1.907 (7)
Li1—O2A^{iii}	1.955 (7)	Li1A—O2^{v}	1.940 (7)
Li1—O3	1.860 (11)	Li1A—O3A	1.886 (7)
Li1—O4A^{iv}	1.908 (7)	O4—Li1A	1.937 (9)
Li1—O4′	1.86 (5)	Li1A—O3′	1.91 (3)
The O—Li—O angles			
O—Li—O angle	∠ (°)	O—Li—O angle	∠ (°)
O1A—Li1A—O2^{v}	119.5 (4)	O1—Li1—O2A^{iii}	116.2 (4)
O1A—Li1A—O3'	101.0 (10)	O3—Li1—O1	107.4 (5)
O1A—Li1A—O4	105.9 (4)	O3—Li1—O2A^{iii}	107.3 (4)
O3A—Li1A—O1A	113.5 (4)	O3—Li1—O4A^{iv}	108.5 (5)
O3A—Li1A—O2^{v}	101.6 (3)	O4A^{iv}—Li1—O1	112.2 (3)
O3A—Li1A—O3'	119.3 (9)	O4A^{iv}—Li1—O2A^{v}	105.0 (3)
O3A—Li1A—O4	104.9 (4)	O4'—Li1—O1	109.0 (19)
O4—Li1A—O2^{v}	110.6 (4)		
The C—O—Li—O torsion angles			
C—O—Li—O torsion angle	∠ (°)	C—O—Li—O torsion angle	∠ (°)
C6A—O3A—Li1A—O1A	31.5 (14)	C6—O3—Li1—O2A^{iii}	−140 (2)
C6A—O3A—Li1A—O2^{v}	161.1 (10)	C6—O3—Li1—O4A^{iv}	107 (3)
C6A—O3A—Li1A—O3'	−87.3 (16)	C6'—O4'—Li1—O1	38 (7)
C6A—O3A—Li1A—O4	−83.6 (12)	C6'—O4'—Li1—O2A^{iii}	−95 (7)
C6—O3—Li1—O1	−14 (3)	C6'—O4'—Li1—O4A^{iv}	154 (7)

Symmetry code(s): (iii) x+1/2, −y+1/2, −z+1; (iv) x+1, y, z; (v) x−1/2, −y+3/2, −z+1.

Table S7. Selected geometric parameters for Li—O bonds in square grid network of LISPRO(α) at 100 K.

The Li—O bonds			
Li—O bond	*d* (Å)	Li—O bond	*d* (Å)
Li1—O1	1.920 (13)	Li1A—O1A	1.872 (14)
Li1—O2A^{i}	1.892 (14)	Li1A—O2^{i}	1.914 (13)
Li1—O3	1.875 (16)	Li1A—O3A	1.883 (13)
Li1—O4A^{ii}	1.909 (12)	Li1A—O4	1.969 (15)
Li1—O4′	1.90 (4)	Li1A—O3’	1.82 (3)
**The O—Li—O angles**			
O—Li—O angle	∠ (°)	O—Li—O angle	∠ (°)
O1A—Li1A—O2^{i}	119.5 (7)	O2A^{i}—Li1—O1	117.8 (7)
O1A—Li1A—O3A	113.8 (7)	O2A^{i}—Li1—O4A^{ii}	110.9 (6)
O1A—Li1A—O4	106.9 (7)	O2A^{i}—Li1—O4'	109.3 (15)
O2^{i}—Li1A—O4	110.9 (7)	O3—Li1—O1	105.3 (7)
O3A—Li1A—O2^{i}	102.3 (6)	O3—Li1—O2A^{i}	106.7 (8)
O3A—Li1A—O4	102.2 (7)	O3—Li1—O4A^{ii}	108.3 (7)
O3'—Li1A—O1A	99.8 (14)	O4A^{ii}—Li1—O1	107.4 (6)
O3'—Li1A—O3A	113.5 (14)	O4'—Li1—O1	113.3 (15)
**The C—O—Li—O torsion angles**			
C—O—Li—O torsion angle	∠ (°)	C—O—Li—O torsion angle	∠ (°)
C1A—O1A—Li1A—O2^{i}	145 (3)	C6'—O3'—Li1A—O2^{i}	6 (11)
C1A—O1A—Li1A—O3A	−94 (3)	C6'—O3'—Li1A—O3A	−107 (10)
C1A—O1A—Li1A—O3'	28 (4)	C6—O3—Li1—O1	−140 (3)
C1A—O1A—Li1A—O4	18 (3)	C6—O3—Li1—O2A^{i}	−14 (3)
C6A—O3A—Li1A—O1A	16 (2)	C6—O3—Li1—O4A^{ii}	105 (3)
C6A—O3A—Li1A—O2^{i}	146.0 (16)	C6'—O4'—Li1—O1	−94 (5)
C6A—O3A—Li1A—O3'	−98 (2)	C6'—O4'—Li1—O2A^{i}	40 (6)
C6A—O3A—Li1A—O4	−99.0 (19)	C6'—O4'—Li1—O4A^{ii}	154 (5)
C6'—O3'—Li1A—O1A	132 (10)		

Symmetry code(s): (i) −*x*+1, *y*+1/2, −*z*+1; (ii) *x*+1, *y*, *z*.

Table S8. Selected geometric parameters for Li—O bonds in square grid network of LISPRO(α) at 298 K.

The Li—O bonds			
Li—O bond	*d* (Å)	Li—O bond	*d* (Å)
Li1—O1	1.912 (17)	Li1A—O1A	1.886 (19)
Li1—O2A^{i}	1.913 (17)	Li1A—O2^{i}	1.926 (18)
Li1—O3	1.841 (19)	Li1A—O3A	1.886 (15)
Li1—O4A^{ii}	1.919 (15)	Li1A—O4	1.919 (19)
Li1—O4′	1.93 (4)	Li1A—O3’	1.85 (4)
**The O—Li—O angles**			
O—Li—O angle	∠ (°)	O—Li—O angle	∠ (°)
O1A—Li1A—O2^{i}	118.4 (9)	O1—Li1—O4A^{ii}	107.1 (9)
O1A—Li1A—O3A	112.1 (8)	O1—Li1—O4'	115.0 (17)
O1A—Li1A—O4	108.3 (10)	O2A^{i}—Li1—O4A^{ii}	110.5 (7)
O3A—Li1A—O2^{i}	101.6 (8)	O2A^{i}—Li1—O4'	109.0 (16)
O3A—Li1A—O4	104.6 (9)	O3—Li1—O1	107.5 (9)
O3'—Li1A—O1A	101.0 (15)	O3—Li1—O2A^{i}	104.8 (10)
O3'—Li1A—O3A	118.9 (16)	O3—Li1—O4A^{ii}	110.1 (10)
O4—Li1A—O2^{i}	110.9 (9)	O4A^{ii}—Li1—O4'	96.5 (12)
O1—Li1—O2A^{i}	116.9 (8)		
**The C—O—Li—O torsion angles**			
C—O—Li—O torsion angle	∠ (°)	C—O—Li—O torsion angle	∠ (°)
C1A—O1A—Li1A—O2^{i}	147 (3)	C6A—O3A—Li1A—O4	−97 (3)
C1A—O1A—Li1A—O3A	−96 (3)	C6'—O3'—Li1A—O1A	137 (11)
C1A—O1A—Li1A—O3'	32 (4)	C6'—O3'—Li1A—O2^{i}	13 (12)
C1A—O1A—Li1A—O4	19 (4)	C6'—O3'—Li1A—O3A	−100 (11)
C6A—O3A—Li1A—O1A	20 (3)	C6—O3—Li1—O1	−144 (4)
C6A—O3A—Li1A—O2^{i}	147 (2)	C6—O3—Li1—O2A^{i}	−19 (5)
C6A—O3A—Li1A—O3'	−97 (3)	C6—O3—Li1—O4A^{ii}	99 (4)

Symmetry code(s): (i) −*x*+1, *y*+1/2, −*z*+1; (ii) *x*+1, *y*, *z*.

Table S9. Selected geometric parameters for Li—O bonds in square grid network of L4MPRO(α) at 100 K.

The Li—O bonds			
Li—O bond	*d* (Å)	Li—O bond	*d* (Å)
Li1—O1	1.922 (3)	Li1—O3	1.852 (3)
Li1—O2^{vi}	1.958 (3)	Li1—O4^{vii}	1.907 (3)
The O—Li—O angles			
O—Li—O angle	∠ (°)	O—Li—O angle	∠ (°)
O1—Li1—O2^{vi}	111.59 (14)	O3—Li1—O4^{vii}	122.60 (15)
O3—Li1—O1	109.49 (15)	O4^{iv}—Li1—O1	109.63 (15)
O3—Li1—O2^{vi}	99.53 (14)	O4^{iv}—Li1—O2^{vi}	103.17 (14)
The C—O—Li—O torsion angles			
C—O—Li—O torsion angle	∠ (°)	C—O—Li—O torsion angle	∠ (°)
C6—O3—Li1—O1		C6—O3—Li1—O4^{vii}	
C6—O3—Li1—O2^{vi}			

Symmetry code(s): (vi) −*x*, *y*−1/2, −*z*+3/2; (vii) *x*−1, *y*, *z*.

Table S10. Selected geometric parameters for Li—O bonds in square grid network of L4MPRO(α) at 293 K.

The Li—O bonds			
Li—O bond	*d* (Å)	Li—O bond	*d* (Å)
Li1—O1	1.924 (9)	Li1—O3	1.840 (8)
Li1—O2^{vi}	1.971 (9)	Li1—O4^{vii}	1.925 (8)
The O—Li—O angles			
O—Li—O angle	∠ (°)	O—Li—O angle	∠ (°)
O1—Li1—O2^{vi}	111.2 (4)	O3—Li1—O2^{vi}	100.1 (4)
O1—Li1—O4^{vii}	109.7 (4)	O3—Li1—O4^{vii}	122.6 (4)
O3—Li1—O1	109.9 (4)	O4^{iv}—Li1—O2^{vi}	102.4 (4)
The C—O—Li—O torsion angles			
C—O—Li—O torsion angle	∠ (°)	C—O—Li—O torsion angle	∠ (°)
C6—O3—Li1—O1	−113.8 (5)	C6—O3—Li1—O4^{vii}	16.7 (5)
C6—O3—Li1—O2^{vi}	129.2 (5)		

Symmetry code(s): (vi) −*x*, *y*−1/2, −*z*+3/2; (vii) *x*−1, *y*, *z*.

Table S11. Selected geometric parameters for Li—O bonds in square grid network of L4MPRO(β) at 100 K.

The Li—O bonds			
Li—O bond	*d* (Å)	Li—O bond	*d* (Å)
Li1—O1	1.937 (4)	Li1—O3	1.865 (4)
Li1—O2^{viii}	1.999 (4)	Li1—O4^{ix}	1.903 (4)
The O—Li—O angles			
O—Li—O angle	∠ (°)	O—Li—O angle	∠ (°)
O1—Li1—O2^{viii}	105.30 (18)	O3—Li1—O4^{ix}	126.5 (2)
O3—Li1—O1	112.41 (18)	O4^{iv}—Li1—O1	102.01 (16)
O3—Li1—O2^{viii}	109.92 (17)	O4^{iv}—Li1—O2^{viii}	98.24 (16)
The C—O—Li—O torsion angles			
C—O—Li—O torsion angle	∠ (°)	C—O—Li—O torsion angle	∠ (°)
C6—O3—Li1—O1	-118.3 (7)	C6—O3—Li1—O4^{ix}	115.9 (7)
C6—O3—Li1—O2^{viii}	-1.4 (7)		

Symmetry code(s): (viii) −*x*, *y* −1/2, −*z*; (ix) *x* − 1, *y*, *z*.

Table S12. Selected geometric parameters for Li—O bonds in square grid network of L4MPRO(β) at 298 K.

The Li—O bonds			
Li—O bond	*d* (Å)	Li—O bond	*d* (Å)
Li1—O1	1.929 (13)	Li1—O3	1.886 (10)
Li1—O2^{viii}	2.000 (12)	Li1—O4^{ix}	1.896 (11)
The O—Li—O angles			
O—Li—O angle	∠ (°)	O—Li—O angle	∠ (°)
O1—Li1—O2^{viii}	105.5 (5)	O3—Li1—O4^{ix}	127.2 (6)
O3—Li1—O1	111.7 (6)	O4^{iv}—Li1—O1	103.0 (5)
O3—Li1—O2^{viii}	108.7 (5)	O4^{iv}—Li1—O2^{viii}	98.4 (5)
The C—O—Li—O torsion angles			
C—O—Li—O torsion angle	∠ (°)	C—O—Li—O torsion angle	∠ (°)
C6—O3—Li1—O1	−102 (3)	C6—O3—Li1—O4^{ix}	131 (2)
C6—O3—Li1—O2^{viii}	14 (3)		

Symmetry code(s): (viii) −*x*, *y* −1/2, −*z*; (ix) *x* − 1, *y*, *z*.

Table S13. Selected geometric parameters for Li—O bonds in square grid network of L4MPRO(γ) at 100 K.

The Li—O bonds			
Li—O bond	*d* (Å)	Li—O bond	*d* (Å)
Li1—O1	1.935 (8)	Li1A—O2^{x}	1.967 (8)
Li1—O1A	1.940 (8)	Li1A—O2A^{xi}	1.964 (8)
Li1—O3	1.884 (8)	Li1A—O4^{xii}	1.879 (7)
Li1—O3A	1.886 (8)	Li1A—O4A	1.851 (8)
The O—Li—O angles			
O—Li—O angle	∠ (°)	O—Li—O angle	∠ (°)
O2A^{xi}—Li1A—O2^{x}	115.4 (3)	O1—Li1—O1A	115.4 (4)
O4A—Li1A—O2A^{xi}	111.2 (4)	O3A—Li1—O1A	99.6 (4)
O4A—Li1A—O2^{x}	102.6 (4)	O3A—Li1—O1	116.4 (4)
O4A—Li1A—O4^{xii}	112.8 (4)	O3—Li1—O1A	116.3 (4)
O4^{xii} —Li1A—O2A^{xi}	103.2 (4)	O3—Li1—O1	100.3 (4)
O4^{xii} —Li1A—O2^{x}	112.0 (4)	O3—Li1—O3A	109.5 (4)
The C—O—Li—O torsion angles			
C—O—Li—O torsion angle	∠ (°)	C—O—Li—O torsion angle	∠ (°)
C6—O3—Li1—O1A	8.6 (10)	C6A—O4A—Li1A—O4^{xii}	127.8 (5)
C6—O3—Li1—O1	133.8 (7)	O1A—Li1—O3A—C6A	132.0 (7)
C6—O3—Li1—O3A	−103.3 (8)	O1—Li1—O3A—C6A	7.2 (10)
C6A—O4A—Li1A—O2A^{xi}	12.4 (7)	O3—Li1—O3A—C6A	−105.5 (7)
C6A—O4A—Li1A—O2^{x}	−111.5 (5)		

Symmetry code(s): (x) −*x*+1, *y*−1/2, −*z*+1; (xi) −*x*+1, *y*+1/2, −*z*+1; (xii) *x*+1, *y*, *z*.

Table S14. Selected geometric parameters for Li—O bonds in square grid network of L4MPRO(γ) at 298 K.

The Li—O bonds			
Li—O bond	*d* (Å)	Li—O bond	*d* (Å)
O1—Li1	1.952 (14)	Li1A—O2^{x}	1.989 (14)
Li1—O1A	1.958 (14)	Li1A—O2A^{xi}	1.988 (13)
Li1—O3	1.894 (11)	Li1A—O4^{xii}	1.873 (11)
Li1—O3A	1.886 (11)	Li1A—O4A	1.858 (12)
The O—Li—O angles			
O—Li—O angle	∠ (°)	O—Li—O angle	∠ (°)
O2A^{xi}—Li1A—O2^{x}	113.4 (4)	O1—Li1—O1A	115.4 (5)
O4A—Li1A—O2A^{xi}	110.3 (7)	O3A—Li1—O1A	100.6 (6)
O4A—Li1A—O2^{x}	104.3 (6)	O3A—Li1—O1	114.8 (7)
O4A—Li1A—O4^{xii}	113.2 (5)	O3A—Li1—O3	112.9 (5)
O4^{xii}—Li1A—O2A^{xi}	105.1 (6)	O3—Li1—O1A	114.7 (7)
O4^{xii}—Li1A—O2^{x}	110.7 (7)	O3—Li1—O1	99.3 (6)
The C—O—Li—O torsion angles			
C—O—Li—O torsion angle	∠ (°)	C—O—Li—O torsion angle	∠ (°)
C6—O3—Li1—O1A	14.2 10)	C6A—O4A—Li1A—O4^{xii}	129.0 (10)
C6—O3—Li1—O1	137.8 (11)	O1A—Li1—O3A—C6A	128.8 (10)
C6—O3—Li1—O3A	-100.2 (11)	O1—Li1—O3A—C6A	4.3 (14)
C6A—O4A—Li1A—O2A^{xi}	11.6 (8)	O3—Li1—O3A—C6A	-108.5 (11)
C6A—O4A—Li1A—O2^{x}	-110.5 (8)		

Symmetry code(s): (x) −*x*+1, *y*−1/2, −*z*+1; (xi) −*x*+1, *y*+1/2, −*z*+1; (xii) *x*+1, *y*, *z*.
